# European robin cryptochrome 4a does not bind 8-HDF or MTHF as second antenna chromophores

**DOI:** 10.1016/j.isci.2026.118094

**Published:** 2026-11-06

**Authors:** Alisha Bhanu Pattani Ameerjan, Bahareh Dabirmanesh, Jonathan Hungerland, Takaoki Kasahara, Rabea Bartoelke, Glen Dautaj, Ghazaleh Saberamoli, Jessica Schmidt, Jingjing Xu, Ilia A. Solov’yov, Karl-Wilhelm Koch, Henrik Mouritsen

**Affiliations:** 1 Neurosensorics/Animal Navigation, Institute of Biology and Environmental Sciences, Carl von Ossietzky Universität Oldenburg, Carl-von-Ossietzky-Str. 9-11, 26129 Oldenburg, Germany; 2 Department of Neuroscience, Division of Biochemistry, Carl von Ossietzky Universität Oldenburg, Carl-von-Ossietzky-Str. 9-11, 26129 Oldenburg, Germany; 3 Institute of Physics, Carl von Ossietzky Universität, Oldenburg, Carl-von-Ossietzky-Str. 9-11, 26129 Oldenburg, Germany; 4 Research Centre for Neurosensory Sciences, Carl von Ossietzky Universität Oldenburg, Oldenburg, Carl-von-Ossietzky-Str. 9-11, 26129 Oldenburg, Germany; 5 Department of Biochemistry and Molecular Biology, University of Southern Denmark, Odense, Campusvej 55, 5230 Odense M, Denmark; 6 Centre for Nanoscale Dynamics (CENAD), Carl von Ossietzky Universität Oldenburg, Oldenburg, Ammerländer Heerstr. 114-118, 26129 Oldenburg, Germany; 7 Department of Biochemistry, Faculty of Biological Sciences, Tarbiat Modares University, Tehran, Iran

**Keywords:** cryptochrome 4a, magnetoreception, antenna chromophores, MTHF, 8-HDF

## Abstract

Cryptochromes and photolyases are blue-light-sensitive flavoproteins that generally bind flavin adenine dinucleotide (FAD) and have distinct functions. Cryptochrome 4a (CRY4a) expressed in the double-cone photoreceptors of migratory songbirds such as the European robin (*Erithacus rubecula*) is the hypothesized primary sensor for avian magnetoreception. In addition to FAD, many photolyases and some cryptochromes bind antenna chromophores such as 8-hydroxy-5-deazaflavin (8-HDF) or 5,10-methenyltetrahydrofolate (MTHF). Here, we investigated whether *Erithacus rubecula* cryptochrome 4a (*Er*CRY4a) also binds 8-HDF and/or MTHF. 8-HDF binding was tested by co-expressing *Er*CRY4a with 8-HDF synthase. 8-HDF bound the positive control *Xenopus laevis* 6-4 photolyase (*Xl*6-4PL), but not *Er*CRY4a. For MTHF, the positive control *Escherichia coli* DNA photolyase (*Ec*DPL) was purified with both MTHF and FAD, whereas *Er*CRY4a showed only FAD binding. Isothermal titration calorimetry also confirmed that MTHF does not bind *Er*CRY4a. Furthermore, computational analysis revealed no binding-site similarity between *Er*CRY4a and MTHF/8-HDF-binding proteins, indicating a non-conserved pocket.

## Highlights

• *Er*CRY4a shows no detectable binding of 8-HDF or MTHF antenna chromophores
• *X. laevis* 6-4 photolyase validates 8-HDF binding under the tested conditions
• *E. coli* DNA photolyase validates MTHF binding under the tested conditions
• Computational analyses reveal barriers to 8-HDF and MTHF binding in *Er*CRY4a

## Graphical abstract

**Figure:**
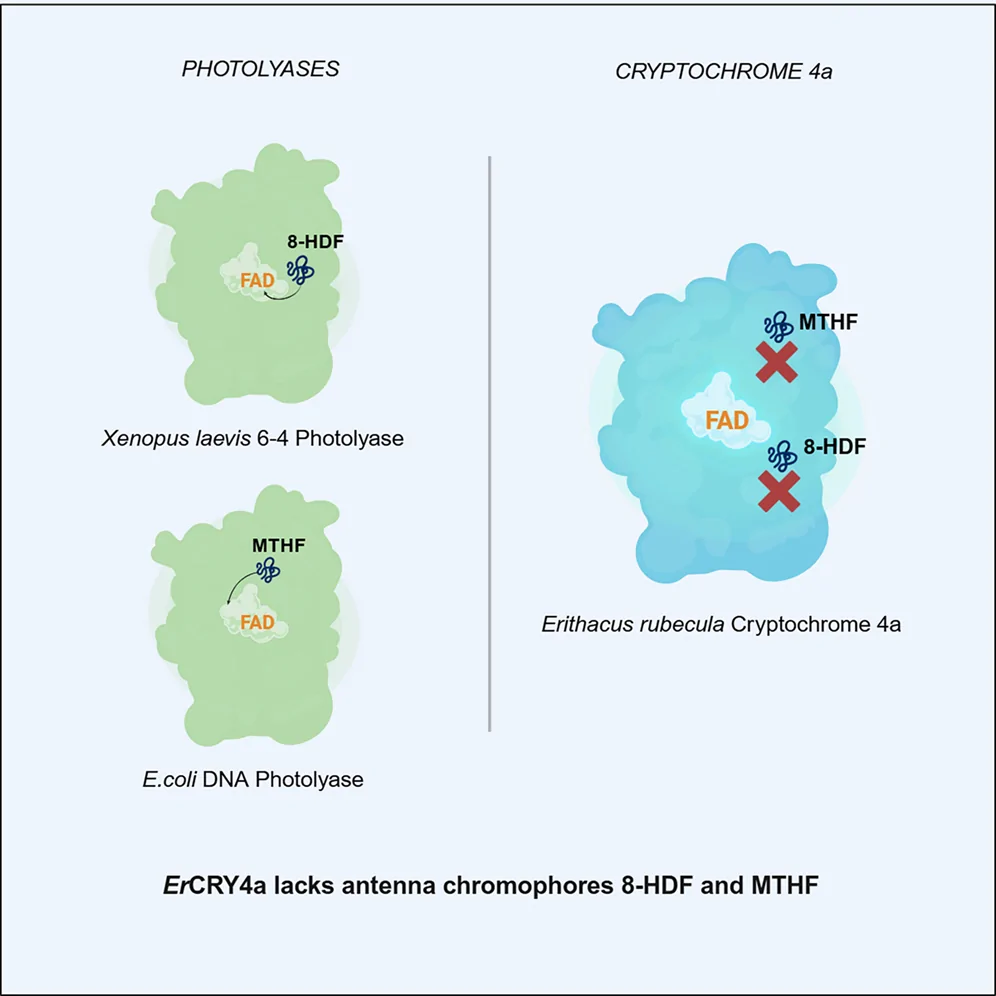

## Teaser

Biochemistry; Structural biology; Computer modeling

## Introduction

The ability of animals to sense and utilize the Earth’s magnetic field for navigation during migration is known as magnetoreception. This remarkable capability has been observed in various species, including for instance songbirds, fish, sea turtles, mammals, and insects.^1^^,^^2^^,^^3^^,^^4^^,^^5^^,^^6^^,^^7^^,^^8^^,^^9^^,^^10^ In night-migratory songbirds, the radical-pair mechanism involving cryptochrome 4a (CRY4a) as the primary magnetic sensor is the most prominent magnetoreception hypothesis. The radical-pair hypothesis proposes that light-induced electron transfer reactions generate radical pairs that are sensitive to weak magnetic fields.^11^^,^^12^^,^^13^ CRY4a is a light-sensitive protein expressed in the double-cone photoreceptors in the retina of migratory songbirds such as the European robin (*Erithacus rubecula*).^14^^,^^15^^,^^16^ This protein has a catalytic light-sensing chromophore called flavin adenine dinucleotide (FAD), which is located in the C-terminal α-helical domain of a conserved photolyase homology region (PHR domain).^17^^,^^18^

Some members of the cryptochrome/photolyase family of proteins are known to bind additional chromophores, mainly 5,10-methenyltetrahydrofolate (MTHF) and 7,8-dimethyl-8-hydroxy-5-deazaflavin (8-HDF).^19^^,^^20^^,^^21^^,^^22^^,^^23^^,^^24^^,^^25^^,^^26^^,^^27^^,^^28^ These additional chromophores have been reported to act as photo-antennas capturing photons and transferring light energy to FAD via the Förster resonance energy transfer (FRET) mechanism.^19^^,^^20^ However, some class II photolyases do not bind a second antenna pigment. For example, the higher plant *Arabidopsis thaliana* class II photolyase lacks binding to any of the known chromophores.^29^

8-HDF is a deazaflavin derivative that is infrequently synthesized by most host organisms.^30^ The biosynthetic pathways for 8-HDF are largely absent outside a few specific groups of microorganisms, algae, and mosses. Vertebrates can acquire 8-HDF solely through dietary intake, particularly by consuming algae or mosses that naturally produce this molecule.^30^^,^^31^^,^^32^ Co-expression of target proteins with 8-HDF synthase-encoding genes (either *fbiC or cofG and cofH*) enables *E. coli*, which lacks an endogenous 8-HDF biosynthetic pathway, to produce the 8-HDF chromophore *in vivo.*^30^ Two independent studies were conducted on the green alga *Chlamydomonas reinhardtii* animal-like cryptochromes (*Cra*CRY), which function both as a circadian-regulating cryptochrome and as a 6–4 photolyase DNA repair enzyme. These studies showed that the binding of 8-HDF as an antenna chromophore to *Cra*CRY kinetically stabilized the flavin-neutral radical (FADH^⋅^) and the reduced FAD (FADH^−^) state.^20^^,^^21^ Furthermore, in Xenopus laevis 6–4 photolyase (Xl6-4PL), 8-HDF captures photons and transfers light energy to the active fully reduced FADH^−^ and enhances the DNA repair activity of the enzyme.^22^ 8-HDF has also been reported to act as a significant photosensitizer in the photoreactivation process catalyzed by DNA photolyase from *Synechococcus elongatus* (formerly known as *Anacystis nidulans*).^23^^,^^24^ More recently, a second photolyase, *Se*PhrB, was characterized in *S. elongatus* as a prokaryotic 6–4 photolyase belonging to the iron-sulfur bacterial cryptochrome and photolyase (FeS-BCP) family, and it was shown to use 8-HDF as its antenna chromophore.^33^

MTHF is a folate derivative that plays a role in folate metabolism.^34^ It is bound to the N-terminal domain of some cryptochrome and photolyase proteins within a specific binding pocket that is exposed to the solvent,^25^ thereby facilitating exchange of the antenna chromophore, which can lead to its loss during purification.^35^^,^^36^ Because of this lability, *in vitro* reconstitution by incubating purified protein with MTHF is often required to assess MTHF binding.^19^ MTHF has been reported to serve as a key component necessary to transfer energy to FADH^−^, enabling it to enter an excited state and initiate DNA repair in *E. coli* DNA photolyase (*Ec*DPL).^28^^,^^37^ Studies on *Ostreococcus tauri* cryptochrome (*Ot*CPF2) and *Phaeodactylum tricornutum* cryptochrome (*Pt*CryP) have highlighted the versatility of MTHF-FAD interactions in improving light energy utilization and electron transfer.^26^^,^^27^ In addition, a class III CPD photolyase from *Agrobacterium fabrum* (also known as *Agrobacterium tumefaciens*) has been shown to harbor MTHF in a binding site distinct from those observed in *Ec*DPL and *Arabidopsis thaliana* cryptochrome 3 (*At*CRY3).^38^

Xu et al. reported the successful purification and characterization of CRY4a from the European robin (*Er*CRY4a), a migratory songbird.^18^ To date, no second chromophore has been identified in the recombinantly expressed and purified *Er*CRY4a. However, a comprehensive understanding of the function of CRY4a requires investigation of all potential chromophores and their interactions with the protein. In this study, we investigated the potential presence of a second chromophore in *Er*CRY4a. A combination of biochemical, spectroscopic, and computational approaches was used to evaluate whether 8-HDF and/or MTHF binds to *Er*CRY4a. Our results support a model in which *Er*CRY4a operates with FAD, but without 8-HDF or MTHF as antenna chromophores, indicating a functional specialization that sets it apart from most photolyases.

## Results

### ErCRY4a does not bind 8-HDF

We expressed the His-tagged recombinant proteins *Er*CRY4a and *Xl*6-4PL under the same conditions, both with and without 8-HDF synthase co-expression, and we purified them using the identical method. UV-Vis spectroscopic analyses of *Er*CRY4a and *Xl*6-4PL revealed that FAD was present in both. However, a significant difference was observed when comparing the spectral profiles of *Er*CRY4a and *Xl*6-4PL co-expressed with 8-HDF synthase. *Xl*6-4PL co-expressed with 8-HDF synthase exhibited a distinctive spectral signature, characterized by a peak at 440 nm and a shoulder at 420 nm (Figure 1), indicative of 8-HDF binding together with FAD. *Xl*6-4PL is a well-characterized protein that binds to both 8-HDF and FAD and shares high sequence similarity with *Er*CRY4a.^22^ Despite this, *Er*CRY4a co-expressed with 8-HDF synthase did not exhibit the characteristic absorption peak of 8-HDF at 440 nm (Figure 1D).

**Figure 1:**
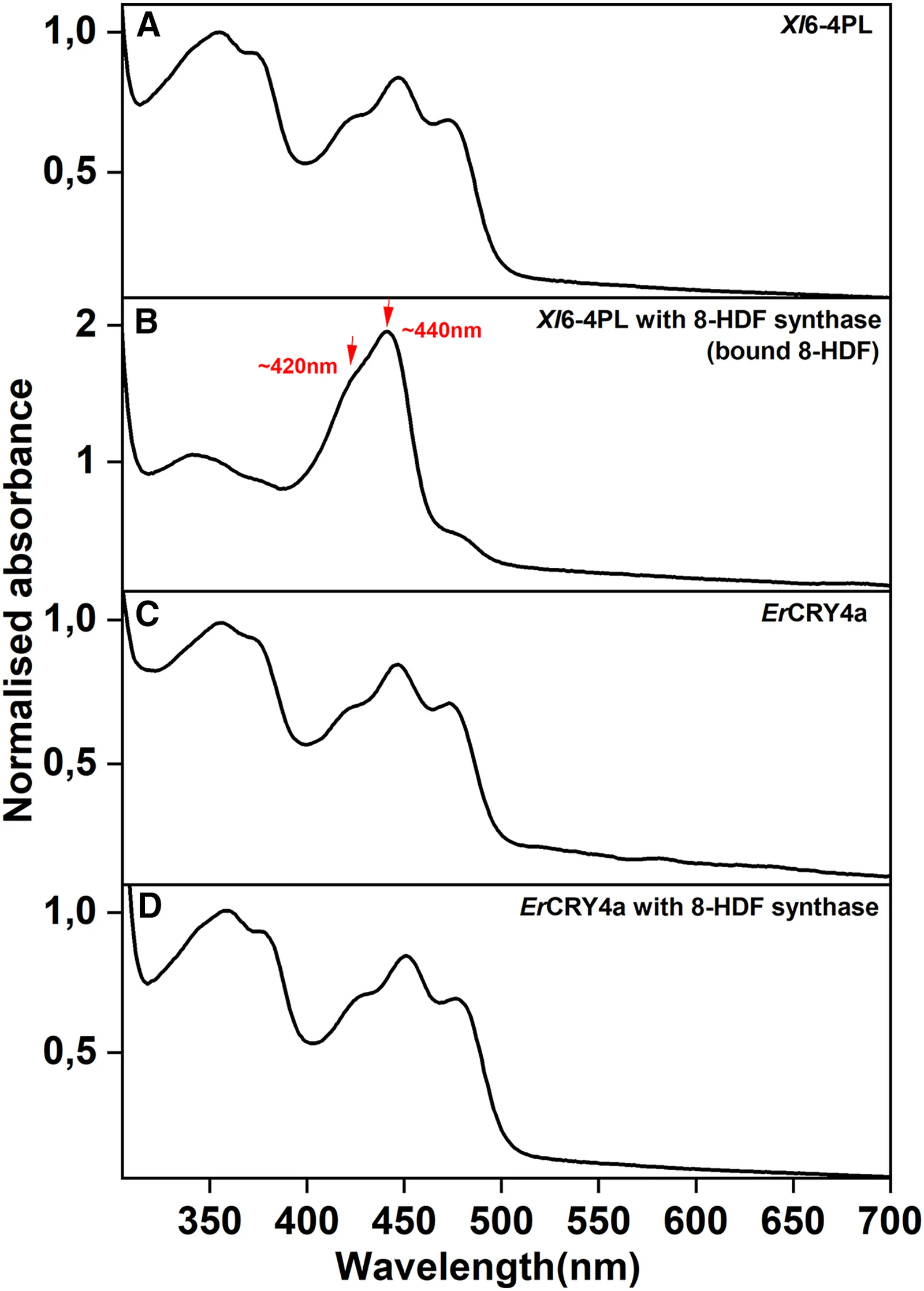
*Er*CRY4a does not bind 8-HDF UV-Vis Spectra of *Erithacus rubecula* cryptochrome 4a (*Er*CRY4a) wild-type and *Xenopus laevis* 6-4 photolyase (*Xl*6-4PL) expressed and purified with and without 8-hydroxy-5-deazaflavin (8-HDF) synthase. Spectra were recorded using 30 μM *Er*CRY4a (for both with and without 8-HDF synthase samples) and 16 μM *Xl*6-4PL (for both with and without 8-HDF synthase samples). Relative absorbance was plotted by normalizing each spectrum to the absorbance at 355 nm. (A) *Xl*6-4PL expressed without 8-HDF synthase shows the characteristic absorption of bound FAD around 350–500 nm. (B) *Xl*6-4PL co-expressed with 8-HDF synthase displays the expected 8-HDF chromophore signature, with a prominent peak at ∼440 nm and a shoulder near 420 nm, in addition to FAD. (C) *Er*CRY4a expressed without 8-HDF synthase exhibits the typical absorption profile of bound FAD. (D) *Er*CRY4a co-expressed with 8-HDF synthase shows no additional absorption features corresponding to 8-HDF, indicating that *Er*CRY4a does not bind 8-HDF under these expression conditions.

### ErCRY4a shows no detectable interaction with MTHF

To investigate the potential interaction between MTHF and *Er*CRY4a, we performed *in vitro* reconstitution with purified *Er*CRY4a and MTHF. *Er*CRY4a (16 μM) was incubated with MTHF (800 μM) for 1 h in the dark at 4°C. To assess the stability of 5,10-MTHF over the complete experimental timescale, absorption spectra of freshly prepared free 5,10-MTHF were recorded at 30-min intervals for 2 h at 4°C under the experimental conditions (Figure S1). Over the course of incubation, a gradual decrease in the characteristic ∼360-nm absorption band was observed, accompanied by progressive spectral changes in the ∼260-nm region, consistent with partial conversion of 5,10-MTHF to 10-formyltetrahydrofolate under neutral conditions, as previously reported.^39^^,^^40^ However, the spectral changes occurred gradually over the experimental timescale, consistent with the low incubation temperature (4°C), which delays the degradation of free 5,10-MTHF under near-neutral conditions.^39^ Thus, although partial interconversion occurred, the persistence of the characteristic 5,10-MTHF absorption band throughout the experimental timescale, together with the 50-fold molar excess of 5,10-MTHF used relative to the protein, indicates that sufficient intact 5,10-MTHF remained available throughout the experiment. UV-Vis spectroscopy analyses of *Er*CRY4a following *in vitro* reconstitution with MTHF revealed absorbance at ∼360 nm (Figure 2B). Free MTHF in solution also absorbs at ∼360 nm (Figure 2D). In contrast, bound MTHF in cryptochromes/photolyases shows a red shift and an absorbance around ∼380 nm.^19^^,^^25^^,^^26^^,^^37^ Since MTHF was present at a 50-fold molar excess over *Er*CRY4a, even complete occupancy of the protein would result in a maximum of 16 μM bound MTHF, whereas the remaining ∼784 μM MTHF would remain free in solution. Consequently, any absorbance arising from protein-bound MTHF would be negligible compared with that of the excess free MTHF and would not be expected to produce a detectable, red-shifted signal prior to removal of unbound ligand. Therefore, the protein-ligand mixture was subjected to centrifugal filtration using a ∼30-kDa molecular-weight cut-off filter to remove free MTHF. Following filtration, neither the absorbance of free MTHF (∼360 nm) nor the characteristic, red-shifted absorbance of protein-bound MTHF (∼380 nm) was detected in the retained protein fraction (Figure 2C), indicating that MTHF was not retained with *Er*CRY4a under the experimental conditions tested.

**Figure 2:**
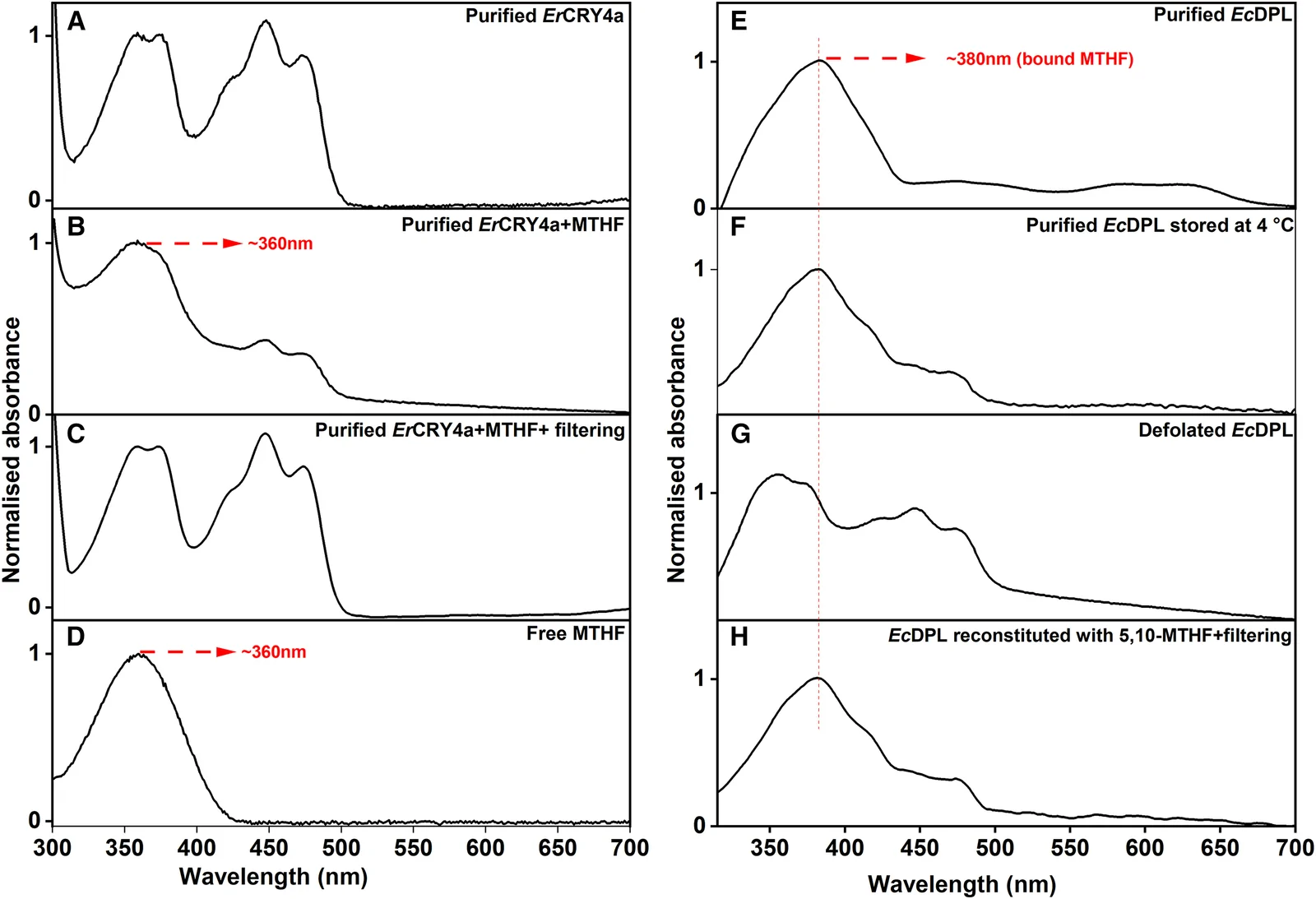
*Er*CRY4a shows no detectable interaction with MTHF (A–H) UV-Visible spectra testing for *Er*CRY4a and 5,10-methenyltetrahydrofolate (MTHF) interactions. Relative absorbance was plotted after normalizing each spectrum to the absorbance at 360 nm for panels A–D and at 380 nm for panels E–H. (A) Purified *Er*CRY4a alone shows the absorption of bound FAD (characteristic shoulder at ∼480 nm). (B) *Er*CRY4a incubated with 800 μM MTHF for 1 h in the dark at 4°C showed the FAD peak along with another peak at ∼360 nm, corresponding to free MTHF. (C) After washing the *Er*CRY4a and MTHF sample through a 30 kDa cut-off filter, the 360 nm peak disappeared and only the absorbance of *Er*CRY4a with bound FAD_ox_ was observed. (D) 800 μM MTHF in buffer (pH 7.5) exhibited a distinct absorbance of free MTHF at ∼360 nm. (E) *E.coli* DNA photolyase (*Ec*DPL) purified under similar conditions displayed the characteristic absorption peak of bound MTHF at ∼380 nm and absorption associated with the neutral semiquinone radical (FADH^⋅^) at ∼580–650 nm. (F) Storage of purified *Ec*DPL at 4°C for 24 h resulted in changes in the visible absorption spectrum consistent with oxidation of the flavin chromophore, while the characteristic MTHF-associated absorption peak at ∼380 nm remained detectable (G) MTHF depletion of *Ec*DPL by deionized-water washing resulted in the loss of the characteristic MTHF-associated absorption peak at ∼380 nm, while the flavin-associated absorption features in the ∼350–500 nm region were retained. (H) *In vitro* reconstitution of MTHF-depleted *Ec*DPL with 5,10-MTHF followed by washing with 30 kDa molecular weight cut-off filter restored the characteristic absorption peak at ∼380 nm, consistent with rebinding of MTHF to *Ec*DPL.

To further validate this result, we expressed and purified a well-characterized MTHF-binding protein, *Ec*DPL,^37^ as a positive control under similar experimental conditions. When we expressed *Ec*DPL under the same conditions we used for *Er*CRY4a, *Ec*DPL was isolated with MTHF bound: we observed the characteristic red shift to ∼380 nm in UV-Vis spectroscopy (Figure 2E). In sharp contrast, *Er*CRY4a expressed and purified under the same experimental conditions was purified without MTHF (Figure 2A). To further validate our experimental approach for assessing MTHF binding, we tested whether MTHF association with *Ec*DPL could be restored after removal and subsequent *in vitro* reconstitution. For this purpose, we removed 5,10-MTHF from purified *Ec*DPL, which we stored at 4°C for 24 h, using the deionized-water washing procedure described by Xu et al.^41^ Upon storage of purified *Ec*DPL at 4°C for 24 h, the visible absorption spectrum changed, consistent with oxidation of the flavin cofactor from the neutral semiquinone FADH^⋅^ state toward oxidized FAD, as previously reported for *Ec*DPL^41^^,^^42^ (Figure 2F). However, the MTHF-associated absorption maximum at ∼380-nm remained detectable. Following MTHF removal, the recovered *Ec*DPL showed a pronounced change of the characteristic MTHF-associated absorption peak in the 380 nm region, while the oxidized flavin-associated absorption features between approximately 300 and 500 nm remained detectable as reported previously (Figure 2G).^42^ We subsequently tested whether MTHF could be reconstituted into MTHF-depleted *Ec*DPL. Defolated *Ec*DPL (16 μM) was incubated with MTHF (800 μM; 50-fold molar excess) for 1 h at 4°C in the dark. Following incubation, unbound MTHF was removed by filtration using a 30-kDa molecular-weight-cutoff filter. UV-Vis analysis of the recovered *Ec*DPL revealed the reappearance of a pronounced absorption peak at approximately 380 nm (Figure 2H), consistent with the reassociation of MTHF with *Ec*DPL.

### Isothermal titration calorimetry (ITC) confirms a lack of detectable MTHF binding to ErCRY4a

To further confirm the absence of an interaction between MTHF and *Er*CRY4a, ITC experiments were performed. Prior to the experiment, the instrument underwent initial calibration using a well-established binding pair with 1 mM Ca^2+^ in the syringe and 100 μM EDTA in the calorimetric cell. Titration yielded exothermic heat pulses (μcal/s), and the corresponding binding isotherm fitted to a one-site binding model demonstrated a distinct saturation point indicative of the binding interactions between the two species (data not shown). Subsequently, the interaction of the purified *Er*CRY4a (ranging from 3 to 30 μM) and MTHF (from 400 to 800μM) was examined at 12°C and 25°C (Figure 3; Figures S2 and S3; Table S1). A control titration of the MTHF ligand into a sodium phosphate buffer at pH 7.5 without *Er*CRY4a present was also conducted. Binding of MTHF to *Er*CRY4a would have resulted in either exothermic or endothermic heat pulses approaching the zero baseline at saturation. Notably, the exothermic heat pulses reached similar amplitudes over a wide concentration range and a molar ratio far above a 1:1 stoichiometry. Furthermore, the exothermic heat pulses of the control titration of MTHF titrated to the buffer (left panels in Figure 3) were higher in all the conditions. The resulting integrated heat data displayed in the lower panel of Figures 3A and 3B did not match a typical binding isotherm. Instead, the integrated heat data expressed in kcal/mol of injectant showed scattered profiles and data points were different from zero. The results of the titration might therefore indicate a weak association of MTHF with the protein surface, but not a permanent interaction with a chromophore-binding pocket. These findings consistently indicate that there was no detectable interaction between MTHF and *Er*CRY4a under the tested conditions.

**Figure 3:**
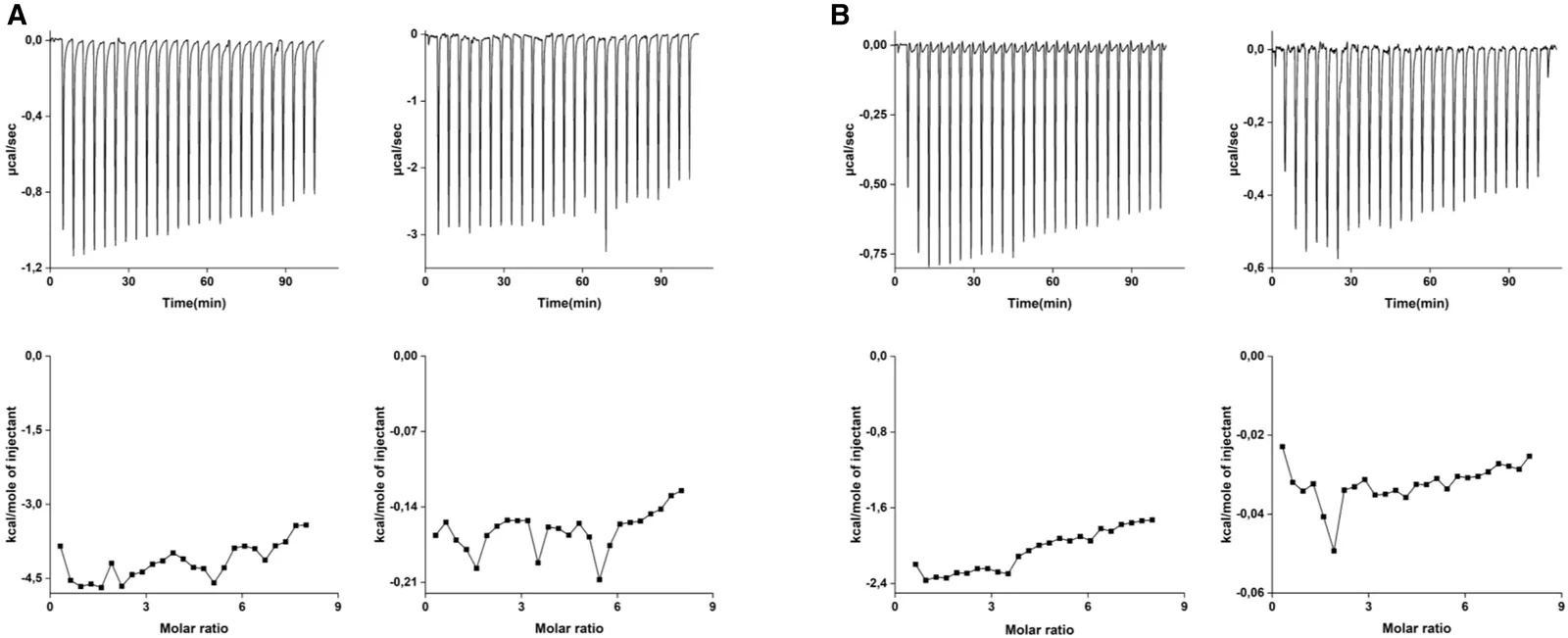
Isothermal titration calorimetry (ITC) confirms a lack of detectable MTHF binding to *Er*CRY4a ITC profiles of *Er*CRY4a titrated with MTHF. (A) 800 μM MTHF was titrated to buffer (left panel) or 16 μM *Er*CRY4a (right panel). Both titrations were performed at 12°C. (B) The same titration of MTHF to buffer (left panel) and 16 μM *Er*CRY4a (right panel) done at 25°C. The data were baseline-adjusted, and the area under the heat pulse curves was analyzed resulting in a plot of kcal/mol of the injectant against the molar ratio (lower parts in (A) and (B)) including the negative controls (MTHF to buffer at both 12°C and 25°C) Similar thermograms were observed across a range of *Er*CRY4a concentrations (3–30 μM) and MTHF concentrations (400–800 μM) (also see Figures S2 and S3; Table S1) indicating no MTHF binding by *Er*CRY4a regardless of the concentration of the ligand.

The combined results from both *in vitro* reconstitution and ITC experiments show that MTHF does not bind to *Er*CRY4a under the conditions tested.

### ErCRY4a shows no predicted interaction with 8-HDF and MTHF *in silico*

As an independent way to assess the potential binding interactions of 8-HDF and MTHF with *Er*CRY4a, the proteins and chromophores were computationally modeled. The fundamental steps of the presented approach are outlined here, while details are provided in the STAR Methods section. At the core of the strategy were the positive controls. We reproduced that 8-HDF binds to *Xl*6-4PL^22^ and that MTHF binds to *Arabidopsis thaliana* cryptochrome 3 (*At*CRY3)^43^ by independently recovering the correct binding pockets of the ligands. While our approach cannot reproduce the exact ligand position from the crystal structure, it can filter out the correct binding pockets from unbiased ensembles of protein-ligand complexes. First, multiple sequence alignments were computed including *Er*CRY4a and all protein structures that were experimentally resolved in the presence of the respective chromophores. The alignment served as an initial probe to estimate where on the protein surface binding could occur and how conserved the amino acid residues were that formed the binding pockets. Afterward, we employed molecular dynamics (MD) simulations to study the protein conformations in the pH range of 7–8, which yielded a diverse set of realistic conformations. Via molecular docking with VINA, a set of optimized locations for the chromophores was found. During this step, the protein was static, but the protein conformations were accounted for by performing the docking against 3000 different protein conformations sampled from MD simulations. In the docking calculations, the chromophores were allowed to explore the whole protein surface (exhaustive docking) and were not restrained to any predetermined binding pocket. Finally, the docked positions were re-evaluated using additional properties other than the VINA score (a binding ΔG estimate). The diffusion score measures how quickly water molecules would be expected to move, if water molecules were found at the optimized location of the chromophore. The diffusion scores were obtained from the MD simulations in which explicit water molecules were present. While the diffusion score is not suitable as a generalizable docking score, it follows a similar idea to SILCS.^44^ The solvent-accessible surface area (SASA) score measures the relative change in solvent accessibility when the chromophore is docked into the optimized location. The entropic barrier score estimates in how many directions the chromophore could move out of its optimized location without encountering anything that blocks its path. To average results from similar positions, we clustered the optimized locations such that positions in which the chromophore is in contact with the same amino acid residues are sorted into the same cluster.

The computational results for the analysis of 8-HDF binding are displayed in Figure 4. An excerpt of the multiple sequence alignment in Figure 4A highlights contacts, primarily but not exclusively formed by hydrogen bonds, between 8-HDF and the surrounding protein structure. The sequence number of *Xl*6-4PL is shown in the top row. The resolved structures *Anacystis nidulans* photolyase (*An*PL), *Thermus thermophilus* photolyase (*Th*PL), *Chlamydomonas reinhardtii* (*Cra*CRY), and *Synechococcus elongatus* 6–4 photolyase (*Sy*PL) share many similarities in their backbone and sidechain contacts to 8-HDF with the exception that *Th*PL has fewer sidechain contacts. Even though *Xl*6-4PL was not structurally resolved in the presence of 8-HDF and can therefore not be categorized in the same way, the occurrence of R51, D101, E103, R109, and K256 suggests that *Xl*6-4PL exhibits binding patterns that are comparable to those of the resolved structures. Notably, *Er*CRY4a contains charged residues of the same type as most 8-HDF-binding proteins at the aligned positions 51, 101, and 103, while lacking those at positions 109 and 256. Therefore, a potential binding motif of 8-HDF in *Er*CRY4a might resemble the one found in *Sy*PL.

**Figure 4:**
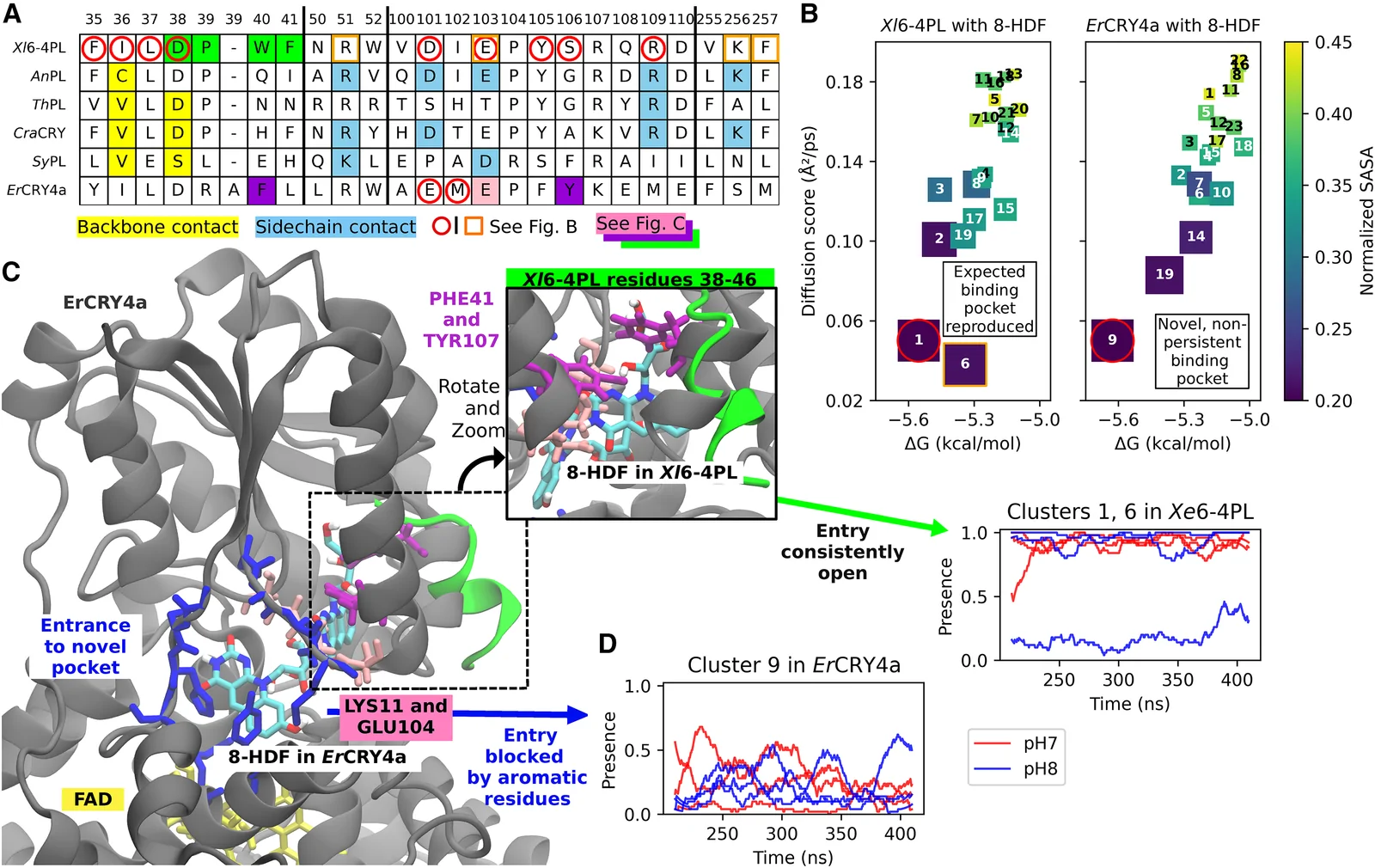
*Er*CRY4a shows no predicted interaction with 8-HDF *in silico* Binding properties of 8-HDF in selected proteins. (A) Partial sequence alignments of the crystallized 8-HDF-binders, *Xl6-4PL* and *Er*Cry4a. The numbers in the first row indicate the residue numbers of *Xl6-4PL*. Protein-ligand interactions observed in crystal structures are color-coded in yellow (backbone contacts) and blue (sidechain contacts). Clusters highlighted in panel B are shape encoded. The amino acids that form the green helix of *Xl*6-4PL in panel C are highlighted in green, and the amino acids of *Er*CRY4a explicitly shown in purple in panel C are highlighted in purple as well. (B) Properties of the docking position clusters. The box sizes were scaled with the sixth power of the entropic barrier score. The text color of the cluster numbers is for readability only. (C) Rendering of the *Er*CRY4a conformation with 8-HDF docked in cluster 9. The 8-HDF position of cluster 1 in *Xl6-4PL* is shown too. The selected residues (Lys11, Glu104, Phe41, Tyr107) from *Er*CRY4a are highlighted. The helix that opens the known binding pocket in *Xl6-4PL* is shown in green. (D) Presence score for the selected clusters over time. Each line corresponds to one Molecular dynamic (MD) simulation.

Exhaustive molecular docking calculations involving 8-HDF were performed, and the analyzed properties are compiled in Figure 4B. The *x* and *y* axes show the ΔG score and the diffusion score, respectively; clusters are indicated by numbers; box colors show the SASA score and box sizes scale with the entropic barrier. The most likely cluster 1 as well as cluster 6 stands out via the optimal scores across all metrics. As indicated by the red circles in the *Xl*6-4PL row of Figure 4A, cluster 1 exhibits most contact residues that are expected to occur based on the sequence alignment. K256, however, is not in contact with 8-HDF when the chromophore is docked in the position of cluster 1. By combining the information from sequence alignment, docking, and re-scoring, the approach therefore reproduced the expected binding pocket of *Xl*6-4PL.

Performing the same exhaustive docking calculations on the *Er*CRY4a protein conformations suggested cluster 9 as a potentially suitable cavity. All scores for cluster 9 are comparable to those of cluster 1 in *Xl*6-4PL. Figure 4C shows a rendering of *Er*CRY4a with 8-HDF docked in the position of cluster 9, which can be found in the middle, right behind the amino acids that form the blue-colored entrance to the binding pocket. However, the blue-colored aromatic residues clearly block the entrance to the pocket. This blockage can be quantified via the presence score for cluster 9. The low presence score suggests that cluster 9 is not an actual binding pocket because it is dynamically inaccessible. It is only suggested as a computational artifact because the docking calculations completely decouple the dynamics of the ligand from the dynamics of the protein. In the same rendering, another possible position of 8-HDF is shown: the location of 8-HDF in cluster 1 of the *Xl*6-4PL docking results. This hypothetical position is buried behind a gray α-helix in the main rendering, and it is more clearly visible in the rotated and zoomed view to the right of the protein rendering in Figure 4C. Amino acid residues 38-46 of *Xl*6-4PL are displayed in the form of a green helix. In contrast to the gray-colored helix of *Er*CRY4a blocking the entrance at the same location, the residues 38-46 helix of *Xl*6-4PL is tilted to the right, which enables entry of 8-HDF into its binding pocket in *Xl*6-4PL. The entrance to clusters 1 or 6 is continuously open in 5 out of 6 replica simulations of *Xl*6-4PL as quantified by the presence score. To sum up, contrary to cluster 9 of *Er*CRY4a, clusters 1 and 6 of *Xl*6-4PL are dynamically accessible.

The results of the computational studies involving the MTHF ligand are summarized in Figure 5. First, the key binding residues were analyzed (Figure 5A). For the *At*CRY3 reference structure, the crystal structure revealed key contacts with residues 149, 150, 417, and 423. The binding patterns of *Deoxyribodipyrimidine* photolyase (*De*PL) and *Arthrospira platensis* photolyase (*Ar*PL) are similar, which supports the assumption that the overall fold of the binding pocket is conserved across these structures. In contrast, *Agrobacterium tumefaciens* photolyase (*Ag*PL) displayed an entirely different binding pattern for MTHF, which was dominated by backbone contacts (Figure 5A, third row). Thus, there are at least two possible and entirely different pocket shapes in *Ag*PL that display tightly packed and structurally verified binding to MTHF. The sequence of *Er*CRY4a lacks the acidic residues at the alignment positions 149 and 150 that coordinate the deeply buried pterin moiety of MTHF in the *At*CRY3 and *Ar*PL structures. Y423, which exhibits π-stacking with the benzene ring of MTHF in *At*CRY3 and *Ar*PL, is also absent in the *Er*CRY4a structure. Differences between the *Ag*PL and *Er*CRY4a sequences suggest that *Ag*PL and *Er*CRY4a do not share a comparable binding pattern.

**Figure 5:**
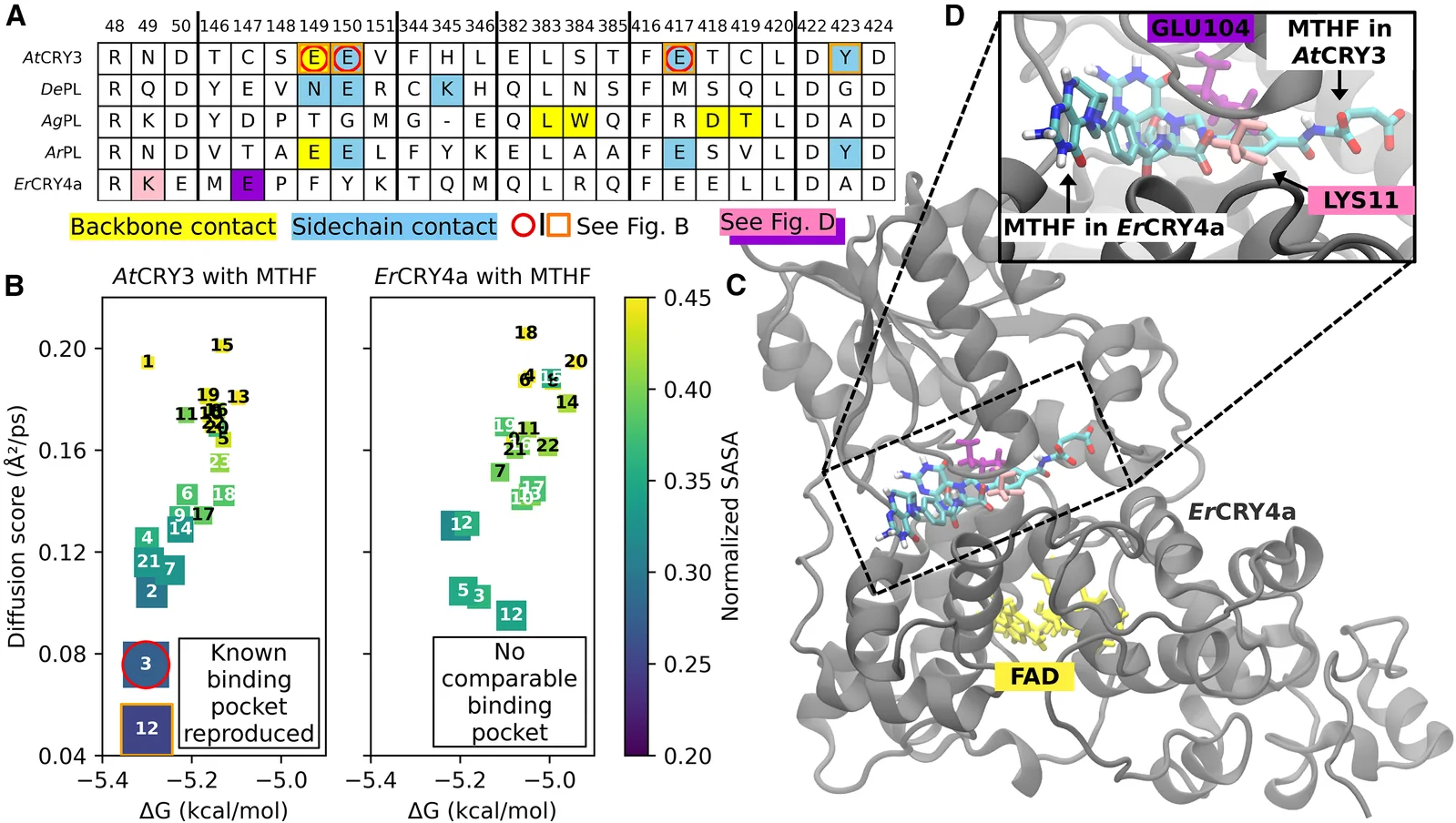
*Er*CRY4a shows no predicted interaction with MTHF *in silico* Binding properties of MTHF in the selected proteins. (A) Sequence alignments *Er*CRY4a compared to *Arabidopsis thaliana (At*CRY3) and other MTHF-binding proteins. The numbers in the first row enumerate the *At*CRY3 residues. Protein-ligand interactions observed in crystal structures are color-coded in yellow (backbone contacts) and blue (sidechain contacts). Clusters highlighted in panel B are shape encoded. The two residues GLU104 and LYS11 of the *Er*CRY4a sequence shown in panel D are highlighted as well. (B) Properties of the docking position clusters. The box sizes were scaled with the sixth power of the entropic barrier score. The text color of the cluster numbers is not significant. (C) Rendering of the *Er*CRY4a conformation with MTHF docked in cluster 3. The MTHF position of cluster 3 in *At*CRY3 is shown in an overlay. (D) Zoom-in of C. The sidechains of *Er*CRY4a Lys11 (Asn49 in *At*CRY3) and *Er*CRY4a Glu104 (Cys147 in *At*CRY3) are shown in pink and purple, respectively.

The results of exhaustively docking MTHF are shown in Figure 5B. For the *At*CRY3 structure, clusters 3 and 12 optimized all analyzed properties simultaneously. The docking positions of cluster 12 were in contact with all experimentally found contact residues (see orange squares in Figures 5A and 5B). For *Er*CRY4a, on the other hand, none of the clusters show pockets with comparable properties. While the ΔG estimate of the best-performing binding pockets in *Er*CRY4a was reduced by only 0.1 kcal/mol compared with correctly identified pockets from the known binders, all other quantities were significantly worse (see Table S2). Via the accumulation of additional binding-related measurements, it can therefore be concluded that MTHF does not bind to *Er*CRY4a. The increased diffusion score, for example, suggests that the local contact surfaces between MTHF and *Er*CRY4a are expected to be subject to large fluctuations and faster water dynamics, which reduces the ligand retention time. Due to the apparent relevance of the diffusion score for our predictions, we performed some test calculations in which the diffusion score replaced electrostatic and hydrogen-bonding contributions from the VINARDO scoring function (see Figure S4). In our formulation, the diffusion score is not directly suitable to be used as a scoring function for molecular docking. However, our results support the value of approaches that utilize the dynamics of small molecules to identify binding pockets.^44^^,^^45^

To investigate the blockage of the hypothetical MTHF-binding pocket, binding positions from cluster 3 of *At*CRY3 and cluster 3 of *Er*CRY4a are shown together in Figures 5C and 5D. The position of MTHF expected in *At*CRY3 served as analogous to place MTHF in *Er*CRY4a, but an ionic bond between Lys11 and either of Glu102 or Glu104 disrupts the access and positioning in the correct place. At least one of those ionic bonds was always formed across all replica simulations. The *Er*CRY4a protein conformations were combined with docked positions of MTHF docked to *At*CRY3, and steric clashes were counted. While the ionic bond between Lys11 and Glu102/Glu104 could in principle reorient to accommodate MTHF, the helix formed by residues 40-47 and the hydrophobic residues Phe106 and Tyr107 sterically prevented the inclusion of MTHF.

## Discussion

Cryptochrome 4a binds the chromophore FAD and is believed to play a crucial role in the ability of birds to detect the Earth’s magnetic field for navigation.^2^^,^^3^^,^^14^^,^^15^^,^^16^^,^^17^^,^^18^^,^^46^ To date, it is unknown whether *Er*CRY4a binds to any other secondary chromophore. Here, we studied the interaction of *Er*CRY4a with two commonly reported chromophores, MTHF and 8-HDF. Co-expression of *Er*CRY4a with 8-HDF synthase did not show the characteristic absorption peak at 440 nm that was otherwise present in the co-expression of the positive control protein *Xl*6-4PL with 8-HDF synthase. The absence of this absorption peak (440 nm) provides compelling evidence that 8-HDF does not bind to *Er*CRY4a. Due to the low solvent accessibility of the expected binding pockets (see results, ‘ErCRY4a shows no predicted interaction with 8-HDF and MTHF in silico’), loss of 8-HDF due to the purification process is unlikely. These results render it very unlikely that 8-HDF binds to *Er*CRY4a, indicating a significant difference in the chromophore-binding properties between *Er*CRY4a and *Xl*6-4PL. For MTHF, both *in vitro* reconstitution experiments and isothermal titration calorimetry (ITC) consistently showed the absence of stable association between MTHF and *Er*CRY4a. Our experimental findings therefore strongly suggest that *Er*CRY4a does not use either of these common chromophores as antennae.

In our computational analysis, we found no similarity at the relevant binding sites between the sequences of *Er*CRY4a and proteins shown to bind MTHF or 8-HDF. This suggests that the binding pocket is not conserved, leaving the possibility of discovering a novel binding conformation. The approach to combine protein ensembles from MD simulations with exhaustive docking, careful preparation of protonation, and water dynamics-informed ranking reproduced the expected binding pockets for 8-HDF in *Xl*6-4PL and MTHF in *At*CRY3. Following this validation, *Er*CRY4a was investigated as a test case. No relevant binding pocket was found for MTHF, whereas a potential novel binding pocket in *Er*CRY4a was found for 8-HDF. The absence of the expected binding pockets was rationalized through analyzing potential steric clashes. A rigid helix formed by residues 40-47 in *Er*CRY4a was found to be mostly responsible for preventing MTHF binding. Furthermore, the protein dynamics suggested that aromatic residues around the entrance to the identified potential novel 8-HDF-binding pocket in *Er*CRY4a effectively hindered 8-HDF from entering it.

The present findings carry important implications for the validity of laboratory-based behavioral tests investigating magnetoreception in migratory birds under the radical-pair hypothesis.^47^^,^^48^^,^^49^^,^^50^^,^^51^ European robins were captured from the wild and subsequently maintained for extended periods under indoor housing conditions prior to behavioral testing. The laboratory diet, which consists primarily of grains, industrially produced mealworms, and vitamin supplements, lacks the algae and bryophytes that synthesize 8-HDF.^30^^,^^31^^,^^32^ Birds kept long-term under these conditions would therefore most likely be depleted of 8-HDF. However, heterologously expressed *Er*CRY4a did not bind 8-HDF, suggesting that it is not a required cofactor for *Er*CRY4a-dependent magnetosensory processes. Accordingly, a deficiency of 8-HDF in captive birds does not compromise laboratory magnetoreception studies, especially because they also orient in behavioral assays. Regarding MTHF, which also appears to be unlikely to be bound with *Er*CRY4a protein, birds should be able to synthesize MTHF from dietary folates. The European robin genome (bEriRub2.2; GCA_903797595.2) contains genes encoding the key enzymes, including MTHFD1, MTHFD2L, SHMT1, and SHMT2. Taken together, these considerations resolve the long-standing concern regarding the nutritional availability of *Er*CRY4a secondary chromophores in behavioral experiments using captive migratory birds.

Our combined experimental and computational results suggest that neither 8-HDF nor MTHF associates with *Er*CRY4a. This is consistent with the broader view that cryptochrome photochemistry is driven primarily by the FAD cofactor and that the core radical-pair mechanism does not require additional antenna chromophores. Previous spectroscopic studies have likewise provided support for FAD-centered photochemistry in cryptochromes, without requiring 8-HDF or MTHF.^18^^,^^52^^,^^53^^,^^54^

### Limitations of the study

Although our study provides strong evidence against the binding of 8-HDF and MTHF to *Er*CRY4a, it does not exclude the possibility that other, yet unidentified, secondary chromophores or cofactors could be involved. Therefore, we suggest that future studies on tissue-derived native *Er*CRY4a should be performed to confirm the absence of additional chromophores or potentially discover novel antenna chromophores.

## Resource availability

### Lead contact

Requests for further information and resources should be directed to and will be fulfilled by the lead contact, Alisha Bhanu Pattani Ameerjan (alisha.bhanu.pattani.ameerjan@uol.de).

### Materials availability

Plasmids generated in this study are available from the lead contact upon request.

### Data and code availability

• All raw and processed data have been deposited at a server of the University of Oldenburg in accordance with the data policy of the Collaborative Research Center SFB 1372 and are publicly available as of the date of publication at https://doi.org/10.57782/IAOQ75 and https://doi.org/10.57782/B5FMYG.
• All original code has been deposited and is publicly available on a server of the University of Oldenburg in accordance with the data policy of the Collaborative Research Center SFB 1372 at https://doi.org/10.57782/B5FMYG.
• Any additional information required to reanalyze the data reported in this paper is available from the lead contact upon request.

## Acknowledgments

This research was supported by the 10.13039/501100001659Deutsche Forschungsgemeinschaft (DFG) through the Collaborative Research Centre “Magnetoreception and Navigation in Vertebrates” (SFB 1372, grant no. 395940726 to IAS, KWK, and HM), through the Transregio Research Centre “HYP∗MOL” (TRR386, grant no. 514664767 to IAS) and through the Excellence Cluster “NaviSense” (EXC 3051, grant no. 533653176 to TK, IAS, HM). The research was also supported by the 10.13039/501100000781European Research Council (ERC) under the European Union’s Horizon 2020 research and innovation program (Grant Agreement No. 810002, Synergy Grant “Quantum Birds”) and the Horizon Europe Programme (HORIZON-MSCA-2024-DN-01 call) (Grant Agreement no. 101225527, MS-RADAM project). The authors also thank the Ministry of Science and Culture of Lower Saxony (Dynamik auf der Nanoskala: Von kohärenten Elementarprozessen zur Funktionalität (DyNano) to IAS). The authors gratefully acknowledge the computing time granted by the Resource Allocation Board and provided on the supercomputer Emmy/Grete at NHR-Nord@Göttingen as part of the NHR infrastructure. The calculations for this research were conducted with computing resources under the project nip00058. We thank Prof. Dr. Lars Oliver Essen (Philipps-Universität Marburg) for kindly providing the pCDFDuet-His₆-FbiC plasmid as a gift. We also thank all members of the Mouritsen group for their support and discussions during this work.

## Author contributions

A.B.P.A. conceived the study, designed and performed the experiments, analyzed and interpreted the data, and wrote and edited the manuscript. B.D. performed ITC experiments together with A.B.P.A., contributed to data analysis, and assisted in writing the manuscript. J.H. performed the computational analyses and contributed to writing the manuscript. T.K. and R.B. conceived parts of the study and provided scientific advice and mentoring. G.D., G.S., and J.S. assisted with protein expression and purification. J.X. provided scientific advice and mentoring throughout the project. I.A.S., K.W.K., and H.M. supervised the project and contributed to data interpretation, manuscript revision, and constructive discussions. All authors read and approved the final manuscript.

## Declaration of interests

The authors declare no competing interests.

## Declaration of generative AI and AI-assisted technologies in the manuscript preparation process

During the preparation of this work, the authors used ChatGPT (OpenAI) to assist with language editing and improving the clarity of the text. The authors reviewed and edited the content as needed and take full responsibility for the content of the publication.

## STAR★Methods

### Key resources table

**Table:** 

REAGENT or RESOURCE	SOURCE	IDENTIFIER
**Bacterial and virus strains**		

*Escherichia coli* BL21(DE3)	Transgen	CD601

**Chemicals, peptides, and recombinant proteins**		

5,10 methenyltetrahydrofolate (MTHF)	Cayman Chemicals	Cat# HY-113046

**Critical commercial assays**		

In-Fusion® HD Cloning Kit	Takara Bio	Cat# 639648

**Deposited data**		

Raw and analysed data	This paper	DARE: https://doi.org/10.57782/IAOQ75
Sequences of recombinant DNA constructs	This paper	DARE: https://doi.org/10.57782/IAOQ75
Molecular dynamics simulation data and analysis	This paper	DARE: https://doi.org/10.57782/B5FMYG

**Oligonucleotides**		

*Xenopus laevis* 6-4 photolyase gene fragment	Integrated DNA technologies	Custom order no# 237184726
*Escherichia coli* DNA photolyase gene fragment	Integrated DNA technologies	Custom order no# 515803424

**Recombinant DNA**		

pCold*Er*CRY4a	Xu et al.^18^	N/A
pCold*Xl*6-4PL	This paper	N/A
pCold*Ec*DPL	This paper	N/A
pCDFDuet-His_6_*FbIC*	Prof. Dr. Lars Oliver Essen	N/A

**Software and algorithms**		

Original code	This paper	DARE: https://doi.org/10.57782/B5FMYG
Origin 2022	OriginLab	https://www.originlab.com/origin
Microcal ITC	Malvern Panalytical/MicroCal	https://www.malvernpanalytical.com/en/products/product-range/microcal-range/microcal-itc-range
OpenFold	Ahdritz et al.^55^	https://github.com/aqlaboratory/openfold
NAMD 3.0	Phillips et al.^56^	https://www.ks.uiuc.edu/Development/Download/download.cgi?PackageName=NAMD
VMD 1.9.4	Humphrey et al.^57^	https://www.ks.uiuc.edu/Development/Download/download.cgi?PackageName=VMD
Meeko	Santos-Martins et al.^58^	https://github.com/forlilab/meeko
AutoDock VINA 1.2.0	Trott and Olson^59^	https://vina.scripps.edu/downloads/
Gaussian 16	Frisch et al.^60^	https://gaussian.com/citation_a03/

### Experimental model and study participant details

#### *Escherichia coli* BL21(DE3)

*E. coli* BL21(DE3) were used as the protein expression host for all recombinant constructs. Competent cells were prepared in-house using standard CaCl_2_/MgCl_2_ methods and stored at -80 °C as aliquots for transformation. Cells were grown in LB medium at 37 °C unless otherwise stated.

### Method details

#### Molecular cloning of plasmids

A pCold*Er*CRY4a plasmid was already available in the lab.^18^ The full-length coding sequence for *Xl*6-4PL (GenBank: AB042255.1) and *Ec*DPL (GenBank: K01299.1) was synthesized by Integrated DNA Technologies (IDT) with flanking sequences (5′–ACGGCGGTTCCTACTAGT–3′ at the N-terminus and 5′–CCCGAGGGATCCGAATT–3′ at the C-terminus) to enable seamless cloning. The DNA fragment was inserted into the pCold expression vector with an N-terminal His tag using the In-Fusion® HD Cloning Kit (Takara, Shiga, Japan).

The plasmid, designated pCDFDuet-His_6_*FbIC* was generously provided by Prof. Dr. Lars Oliver Essen (Philipps-Universität Marburg). This plasmid contained the *Streptomyces coelicolor* (UniProt: Q9KZZ7) *SCO4429* (*fbiC*) gene that enabled the heterologous expression of the 8-HDF synthase in *E. coli*. This approach allowed us to produce this enzyme in a well-characterized and easily manipulatable bacterial host.

#### Expression and purification of ErCRY4a, Xl6-4PL and EcDPL

Competent *E. coli* BL21(DE3) cells were transformed with pCold*Er*CRY4a or pCold*Xl*6-4PL or pCold*Ec*DPL and the transformants were selected on LB plates containing ampicillin (100 μg mL^–1^). A single colony was inoculated into 30 mL of LB media supplemented with ampicillin (100 μg mL^–1^). The overnight culture, incubated at 37 °C, was subsequently inoculated into 700 mL LB medium containing ampicillin (100 μg mL^–1^). The culture was incubated at 37 °C, 200 rpm with agitation until an OD600 of 0.6 was reached. IPTG was added to a final concentration of 10 μM, and the culture was incubated at 15 °C, 160rpm for 44 h. This was then subjected to purification. All purification procedures followed previously established protocols described in Xu et al. (2021) except that 50mM Imidazole was used instead of 20mM, in the wash buffer.^18^

Antenna chromophores that bind during expression remain tightly associated with the protein throughout affinity purification.^61^ For chromophore binding study, *Er*CRY4a and *Xl*6-4PL that are co-expressed with 8-HDF synthase and *Ec*DPL were therefore purified using Ni-NTA affinity chromatography only, as this purification level is sufficient for evaluating chromophore incorporation. And, for a better comparison, *Er*CRY4a and *Xl*6-4PL expressed without 8-HDF synthase were also purified using Ni-NTA affinity chromatography only. For the MTHF binding experiments using *in vitro* reconstitution, *Er*CRY4a was purified using a two-step purification workflow consisting of Ni-NTA affinity chromatography followed by anion exchange chromatography based on the surface charge of the proteins using the ÄKTA pure 25 M system (GE Healthcare, Sweden). Higher protein purity is necessary for *in vitro* reconstitution assays and for downstream biophysical characterization.

#### Co-expression of 8-HDF synthase with ErCRY4a and Xl6-4PL

For co-expression of *Er*CRY4a and *Xl*6-4PL together with the 8-HDF synthase the same expression protocol was used with following modifications: *E. coli* BL21(DE3) competent cells were transformed with pCDFDuet-His_6_*FbIC* and pCold*Er*CRY4a or pCold*Xl*6-4PL, and streptomycin (50 μg mL^–1^) was added in addition to ampicillin in every step, due to the streptomycin resistance present on pCDFDuet-His_6_FbIC.

*Er*CRY4a and *Xl*6-4PL proteins co-expressed with 8-HDF synthase were purified by Ni-NTA affinity chromatography according to the protocol established by Xu et al. (2021) except that 50mM Imidazole was used instead of 20mM, in the wash buffer.^18^

#### Removal of 5,10-MTHF from purified EcDPL by deionized-water washing

MTHF-depleted *Ec*DPL was prepared using the deionized-water washing procedure described by Xu et al. (2006).^41^ 15 mL of 20 μM holo-*Ec*DPL (stored at 4 °C for 24 h) was loaded onto an immobilized nickel-affinity column equilibrated with binding buffer containing 20 mM Tris, 150 mM NaCl and 5 mM imidazole (pH 7.5). The immobilized protein was washed with 30 column volumes of deionized water and subsequently eluted with buffer containing 20 mM Tris, 150 mM NaCl and 300 mM imidazole (pH 7.5). The recovered MTHF-depleted *Ec*DPL was analyzed by UV–Vis absorption spectroscopy using a Cary 60 UV–Vis spectrophotometer (Agilent Technologies, USA) to assess removal of the MTHF-associated absorption feature and retention of the flavin-associated spectral features.

#### *In vitro* reconstitution of ErCRY4a and EcDPL with MTHF

To test for *in vitro* reconstitution, we purified *Er*CRY4a and MTHF-depleted *Ec*DPL (positive control) and diluted both samples to a final protein concentration of 16 μM in 20 mM sodium phosphate buffer (pH 7.5). To assess MTHF (Cayman Chemical) stability over the complete experimental timescale, MTHF was freshly prepared for each experiment in the same buffer used for the protein measurements and added to the protein to a final concentration of 800 μM (50-fold molar excess relative to the protein). To directly monitor the behavior of free MTHF under these experimental conditions, UV–Vis absorption spectra were recorded immediately after preparation and subsequently at 30-min intervals for 2hrs, corresponding to the duration of the experiments. The reconstitution of *Ec*DPL with MTHF was previously demonstrated.^39^^,^^40^^,^^41^^,^^42^^,^^62^^,^^63^^,^^64^ Samples were incubated for 1 h at 4 °C in the dark. Following incubation, unbound MTHF was removed by washing using 30-kDa molecular-weight-cutoff centrifugal filters, with centrifugation at 4,000 × *g* for 7 min at 4 °C for each step. UV–Vis absorption spectra were recorded before and after removal of unbound MTHF using a Cary 60 UV–Vis spectrophotometer (Agilent Technologies, USA) over a wavelength range of 200–800 nm.

#### Isothermal titration calorimetry (ITC) assay

ITC assay was employed to assess the potential binding interactions between *Er*CRY4a and MTHF using a VP-ITC from MicroCal (Malvern panalyical, Northhampton). A detailed step-by-step operation procedure of an ITC experiment was described previously.^65^ For the present application we used an ITC buffer containing 20 mM sodium phosphate at pH 7.5 for preparation of stock solutions of purified *Er*CRY4a and MTHF (Cayman Chemicals). Prior to titration, all solutions were filtered and degassed using a vacuum pump, and both *Er*CRY4a and MTHF were placed in the 20 mM sodium phosphate buffer at pH 7.5 to mitigate potential artifacts arising from buffer mismatch. In each experiment, 1.6 mL of *Er*CRY4a was placed in the calorimetric cell at a final concentration ranging from 3 to 30 μM, depending on the titration. The syringe contained 300 μL of MTHF at a concentration of 400-800 μM. The titration involved 26 injections, beginning with an initial 2 μL injection, followed by 25 injections of 10 μL each. The reference power was set at 10 μcal/sec, the stirrer rate was 300 rpm, and the spacing between the injections was adjusted to allow for complete heat return to the baseline. The experiments were conducted at both 12 °C and 25 °C. Control titrations of MTHF into the buffer were conducted under the same conditions to account for dilution and background heat. Data was analyzed using Microcal ITC and Origin 2022 softwares.

#### MD simulations

In this study, three protein structures were subjected to MD simulations and molecular docking with the target ligands, MTHF and 8-HDF. As a positive control, MTHF-binding *At*CRY3 and 8-HDF-binding *Xl*6-4PL were used. *At*CRY3 has previously been crystallized in the presence of MTHF.^25^ This crystal structure was used for simulations. The other protein structures were obtained as unrelaxed predictions of the OpenFold^55^ implementation of the AlphaFold2^66^ algorithm. Structure predictions relied on the sequences for *Er*CRY4a and *Xl*6-4PL. Accurate structure predictions were possible because OpenFold had access to template structures available through the following PDB IDs: 6FN0 [animal-like Cryptochrome],^67^ 1QNF [bacterial photolyase],^68^ 1TEZ [bacterial photolyase],^69^ 4U63 [bacterial photolyase],^38^ 6PU0 [pigeon cryptochrome 4],^17^ 6PTZ [pigeon cryptochrome 4],^17^ 5ZM0 [animal-like cryptochrome],^67^ 2WQ7 [drosophila 6-4 photolyase],^70^ and 3CVV [drosophila 6-4 photolyase].^70^

The MTHF binding pocket of the *At*CRY3 crystal structure does not involve experimentally unresolved terminal residues. Thus, it was assumed that the exclusion of flexible terminal residues did not change the relevant part of the molecular binding calculations. Additionally, the conformational space of larger flexible parts would not be explored appropriately during the planned simulation time. Excluding the flexible termini of the protein from the simulation therefore avoids artifacts that may arise due to the arbitrary initial position of the involved atoms. The resolved amino acids of the *At*CRY3 crystal structure were used as *a priori* benchmark for sufficient rigidity. After sequence-structure alignment using STAMP,^71^ interfaced through the MultiSeq tool^72^ in VMD,^57^ residues that aligned with the resolved part of *At*CRY3 were retained. These included residues 39-496 for *At*CRY3, residues 1-474 for *Er*CRY4a and residues 1-475 for *Xl*6-4PL. All structures were solvated in a 120 Å cubic simulation box filled with water at a salt-concentration of 0.15 mol/L NaCl. pKa values of the protein residues were estimated with PROPKA3^73^ and two separate structures were prepared according to macroscopic pH values of 7 and 8. Studying multiple protonation states allows the exploration of different protonation-dependent conformations of proteins^74^ and reduces the sensitivity to errors in the protonation state prediction tool. When the results were averaged over both pH values, an experimental pH of 7.5 was modelled.

MD simulations were performed using NAMD3^56^^,^^75^ employing the CHARMM force field^76^^,^^77^^,^^78^ and FAD parameters.^79^^,^^80^^,^^81^ Employing the same settings as described previously,^46^^,^^82^ an initial 20,000 gradient descent (standard numerical optimization algorithm) steps were followed by a 10 ns equilibration simulation using the NPT statistical ensemble with a 1 fs integration time step, followed by 400 ns simulations with a 4fs time step within the NVT statistical ensemble. After 10 ns of NPT equilibration and another 50 ns of RMSD convergence, we still observed some local configurational changes as identified via jumps in the RMSD of a few replicas (see Figure S5). To increase the diversity between samples from different replicas, we excluded the first 210 ns of simulation time from the analysis and docking calculations. Three independent replica simulations were performed for both pH values.

#### Molecular docking

As the first step in ligand preparation, potential tautomer (different protonation states), were explored. Due to the inability of crystallography to capture hydrogen and the high relevance of hydrogen bonds in molecular docking, the molecular protonation pattern should be treated carefully. Possible tautomer were manually identified and optimized in vacuum at the MP2/cc-pVTZ level of theory using the Gaussian16 software.^60^ The energies were then evaluated, including an implicit solvation that solves a Poisson equation using the electronic density (SMD).^83^ For MTHF, the Pteridine molecule was used as a reduced model structure because the protonation of the remaining molecular parts (benzene, carbonylamino, and carboxyl groups) was clear. For 8-HDF, deazaisoallaxine was subjected to calculations to determine the unclear protonation states of 8-HDF.

The ligands were prepared using Meeko^58^ with Gasteiger charges and explicit water molecules (hydrated docking process). Gasteiger charges are derived from topology and ensure a consistent set of partial charges across all molecular conformers. To prepare the receptor structures, one frame every 400 ps was extracted from the production MD simulation and prepared with Meeko while retaining the CHARMM partial charges and maintaining FAD as part of the receptor. A custom script was used to convert the atom types of FAD into Sybyl atom types. To avoid bias in the known binding pockets, exhaustive docking was performed, including the full receptor in the search space. By aligning all structures to the *At*CRY3 crystal structure before docking, it was possible to use the same search space for all receptors and conformations, which resulted in a box of 90 × 65 × 65 Å^3^. Vina^59^ was used for docking with 384 docking attempts (exhaustiveness), a grid spacing of 0.375 Å, a minimum RMSD between the output positions of 2 Å, and an energy range of 10 kcal/mol. In total, resulting in

2pHValues·3replicas·2.5frames/ns=3000conformations

per receptor which were each subjected to 364 docking attempts. With up to 80 docked positions per docking run (some produced less), approximately 480k docked complexes were generated for each receptor-ligand pair. These structures were used to sample the conformational space of possible ligand-docking locations across the protein and were further analyzed. Possible conformations of the ligands were purely explored through docking simulations. No molecular dynamics involving 8-HDF or MTHF were performed.

#### Analysis of docking positions

This section defines all the properties that were employed to analyze, rank, and characterize the binding pockets of the studied proteins.

##### Alignment to known binding proteins

The crystal structures of proteins that included a bound MTHF or 8-HDF ligand were manually inspected, and contacts of the ligand molecules with side chains or backbones of certain amino acid residues were noted separately. Structure-informed sequence alignment using STAMP^71^ was performed with the crystal structures and predicted folds of *Xl*6-4PL and *Er*CRY4a. The aligned sequences were then combined with information on key contacts.

• *At*CRY3 [PDB: 2IJG]^25^
• *De*PL [PDB: 1DNP]^84^
• *Ag*PL [ PDB: 4U63]^38^
• *Ar*PL [PDB: 6KII]^85^

• *An*PL [PDB: 1QNF]^68^
• *Th*PL [PDB: 2J07]^86^
• *Cra*CRY [PDB: 6FN2]^67^
• *Sy*PL [PDB: 8IJY]^87^

##### Clustering

For a given receptor-ligand pair, the ligand docking positions were clustered based on the most dominant contact residues. Contact residues were defined as protein (or FAD) residues that appeared within 4 Å of the ligand binding site. First, the ligand-receptor contact probabilities of all residues were computed. All docked positions where the most probable contact residue appeared to be a contact residue were assigned to cluster 1. Subsequently, the contact probabilities were recomputed using all unassigned docking positions. From the recomputed contact probabilities, all docked positions where the new most probable contact residue was a contact residue were assigned to cluster 2. This procedure was repeated until 95% of all docked ligand positions were assigned to a cluster. All remaining docked positions were assigned to the final cluster.

##### Presence

The presence score for a cluster is given as the fraction of MD simulation frames in which the given cluster was found at least once within the docked positions. For visualization purposes, this property is computed every 0.1 ns but a time-average of a moving window of 5 ns is shown. Such an average is necessary since a cluster can only be either present among the docked positions for a particular simulation frame (1) or not (0), which leads to large fluctuations in the data when showing the presence over time.

##### ΔG

For the binding free energy estimate of ligands’ docked positions, the Vina score was used and termed ΔG throughout.

##### Diffusion

The diffusion scores model the expected dynamics of a docked ligand based on the dynamics of the water molecules from the MD simulation. A diffusion score was assigned to each protein residue; the diffusion score of the ligands’ docked positions was computed as the average diffusion score across the contact residues. To assign the per-residue diffusion scores, the displacements over 200 ps of all water molecules within 4 Å of a residue were computed such that each water molecule within 4 Å contributed a data point. These computations were accumulated into a histogram of displacements for each residue of the protein. For a truly diffusive process, the histogram is expected to approximate a Maxwell distribution profile^88^ with a single diffusion coefficient:

(Equation 1) $$p\left(r;D,\Delta t\right)=\frac{r^{2}}{\sqrt{4\pi {\left(D\Delta t\right)}^{3}}}\exp\left(-\frac{r^{2}}{4D\Delta t}\right)$$

Here, the Maxwell distribution p(r) is a probability distribution of the displacement *r* and depends on the time interval between observations Δt and the diffusion coefficient D. Because the dynamics of bound water molecules represent a sub-diffusive process,^89^^,^^90^ it is generally necessary to fit multiple Maxwell components simultaneously. For two components, the multi-component Maxwell distribution is expressed as

(Equation 2) $$p_{m}\left(r;m_{1},D_{1},D_{2},\Delta t\right)=m_{1}p\left(r;D_{1},\Delta t\right)+m_{2}p\left(r;D_{2},\Delta t\right),\text{with}\;m_{2}=1{-m}_{1}$$

This combination of two Maxwell components allows the separation of the contributions of close but unbound water molecules and close, bound water molecules. For most residues, three components were sufficient to obtain good (R^2^ > 0.9) fit to the histograms of the mean squared water molecule displacement. For the exceptional cases, the histogram was unsteady, and the protein residues were buried, indicating a lack of data points for the histograms and not a lack of model expressiveness. Even in these cases, the fits still agreed (R^2^ > 0.8) with the overall shapes of the histograms. The diffusion score for a protein residue was computed as the average diffusion coefficient $=\sum_{i}m_{i}D_{i}$ .

##### Normalized SASA

For every docked position, the solvent-accessible surface area (SASA) was measured in VMD^57^ using a probe radius of 1.4 Å with the surface restricted such that the contact surface between the protein and the ligand was excluded (protein+ligand as the main selection and using the ligand for the restricted option). The obtained SASA values were then normalized by the surface area obtained when the contact with the protein was ignored.

##### Entropic barrier score

The entropic barrier score is a crude estimate of the size of the exit pathway for the molecule out of the pocket. To understand the approach, one may imagine that all atoms of the ligand emit light rays, and it is measured how many of those are absorbed by the protein. Algorithmically, this is solved by selecting a random atom of the protein at position $\vec{r_{0}}$ and then defining a cylinder of width R that starts at $\vec{r_{0}}$ and points infinitely long into a random direction $\vec{u}$. Subsequently, it is determined whether any atom of the protein lies within the cylinder. Repeating the calculations with many randomly selected ligand atoms and random directions allows convergence towards a score between 0 (no hits) and 1 (every cylinder contains atoms of the protein). To achieve this, one tests whether there are atoms of the protein for which the position vector $\vec{r}$ satisfies the following conditions:

(Equation 3) $${\left|\left|\left(\vec{r}-\vec{r_{0}}\right)\cdot \vec{u}\right|\right|}^{2}<R^{2}$$

(Equation 4) $$\left(r_{i}-r_{0,i}\right)u_{i}\geq 0,\text{for}\;i=\left\{x,y,z\right\}$$

Here, “·” denotes the dot-product. Note that $\vec{u}$ is a normalized vector that is uniformly sampled from the unit sphere. The first condition ensures that the atoms are within the radius of the cylinder, and the second condition ensures that the cylinder begins at position $\vec{r_{0}}$ and stretches to infinity from there. The random atoms and directions were sampled until the change in the hit rate converged below 0.01. The cylinder radius was set to R = 1.5 Å. A high entropic barrier score is expected to be strongly correlated with a high normalized SASA, but the entropic barrier score also allows differentiation between shallow binding pockets (where most light rays will miss) and deeply buried binding pockets (where most light rays will eventually hit a protein atom).

##### Clash probability

The protein folds were overall similar, but the locations of the docked ligands were different. A measurement was desired to count the number of atoms that would clash if the docked position of a chromophore inside a protein was overlayed with a different protein structure. For this purpose, the docked positions of MTHF in *At*CRY3 and 8-HDF in *Xl*6-4PL that were found within the most favorable binding clusters were selected. For each docked ligand position in the reference proteins, it was counted how often a heavy atom from the *Er*CRY4a trajectory was within 1 Å of a heavy atom of the ligand. Using the pre-aligned positions generated for docking preparation, the resulting clashes were directly attributed to differences in the internal coordinates of the proteins. The number of frames showing a clash with any atom of a certain residue was normalized by the total number of simulation frames of the *Er*CRY4a trajectory, resulting in a clash probability for every residue.

### Quantification and statistical analysis

There are no quantification or statistical analyses to include in this study.
