# Supplementary material for: European robin cryptochrome 4a does not bind 8-HDF or MTHF as second antenna chromophores
Source: iScience. 2026 Nov 6;29(11):118094. doi: 10.1016/j.isci.2026.118094 (PMC13769073; doi:10.1016/j.isci.2026.118094)
Supplement: Supplementary file 1 [file mmc1.pdf]

**iScience, Volume *29***

## Supplemental information

# European robin cryptochrome 4a

# does not bind 8-HDF or MTHF

# as second antenna chromophores

**Alisha Bhanu Pattani Ameerjan, Bahareh Dabirmanesh, Jonathan Hungerland, Takaoki Kasahara, Rabea Bartoelke, Glen Dautaj, Ghazaleh Saberamoli, Jessica Schmidt, Jingjing Xu, Ilia A. Solov'yov, Karl-Wilhelm Koch, and Henrik Mouritsen**

**Supplementary Information:**

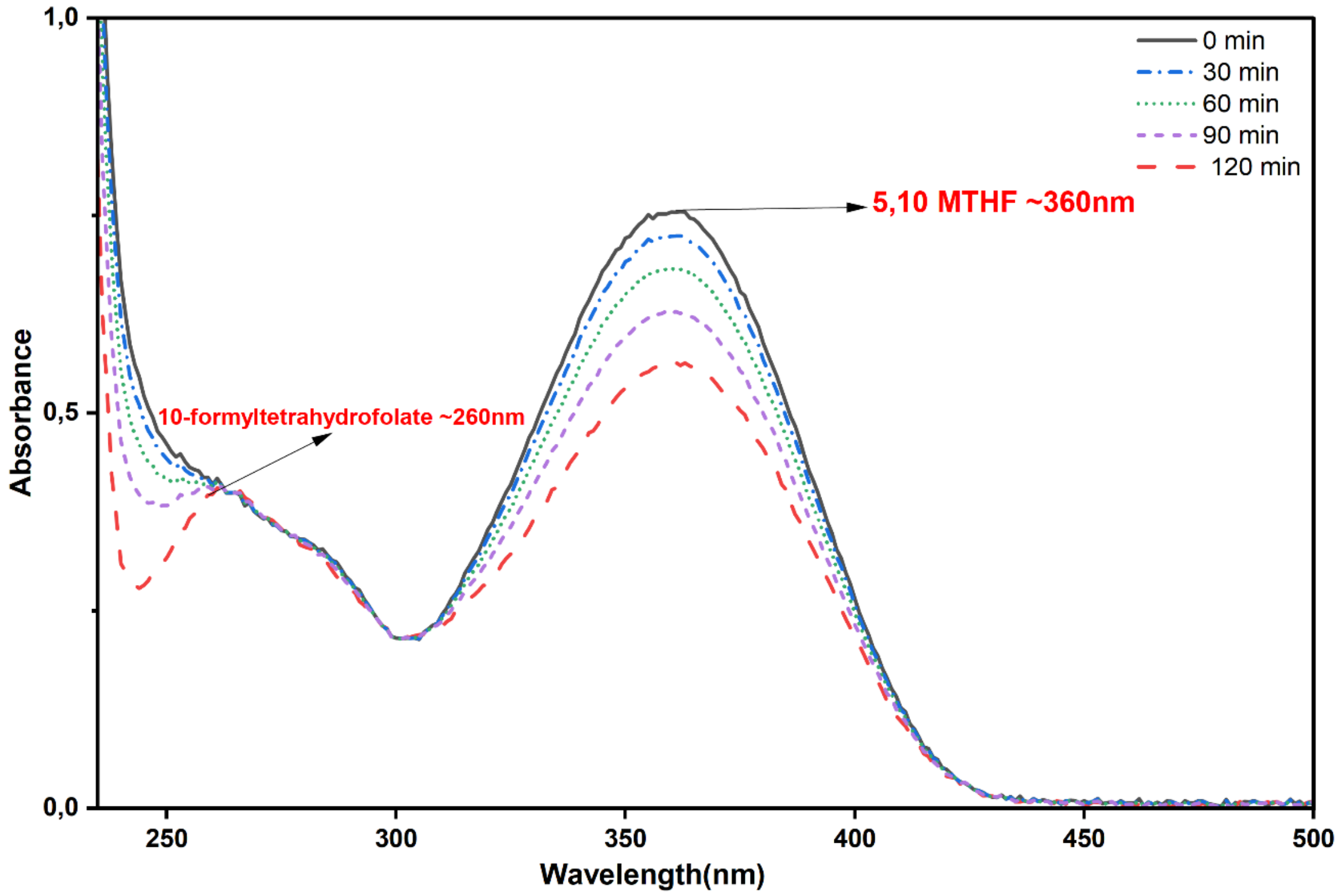


**Figure S1: Stability of free 5,10-methenyltetrahydrofolate (5,10-MTHF) under the experimental conditions, Related to Figure 2.** UV–Vis absorption spectra of freshly prepared free 5,10-MTHF in sodium phosphate buffer (pH 7.5), recorded immediately after preparation and at 30-min intervals for 2 h at 4 °C. A progressive spectral changes in the ~260-nm region was observed over time, consistent with partial conversion of 5,10-MTHF to 10-formyltetrahydrofolate, while the characteristic ~360-nm MTHF absorption band remained clearly detectable throughout the experimental timescale.

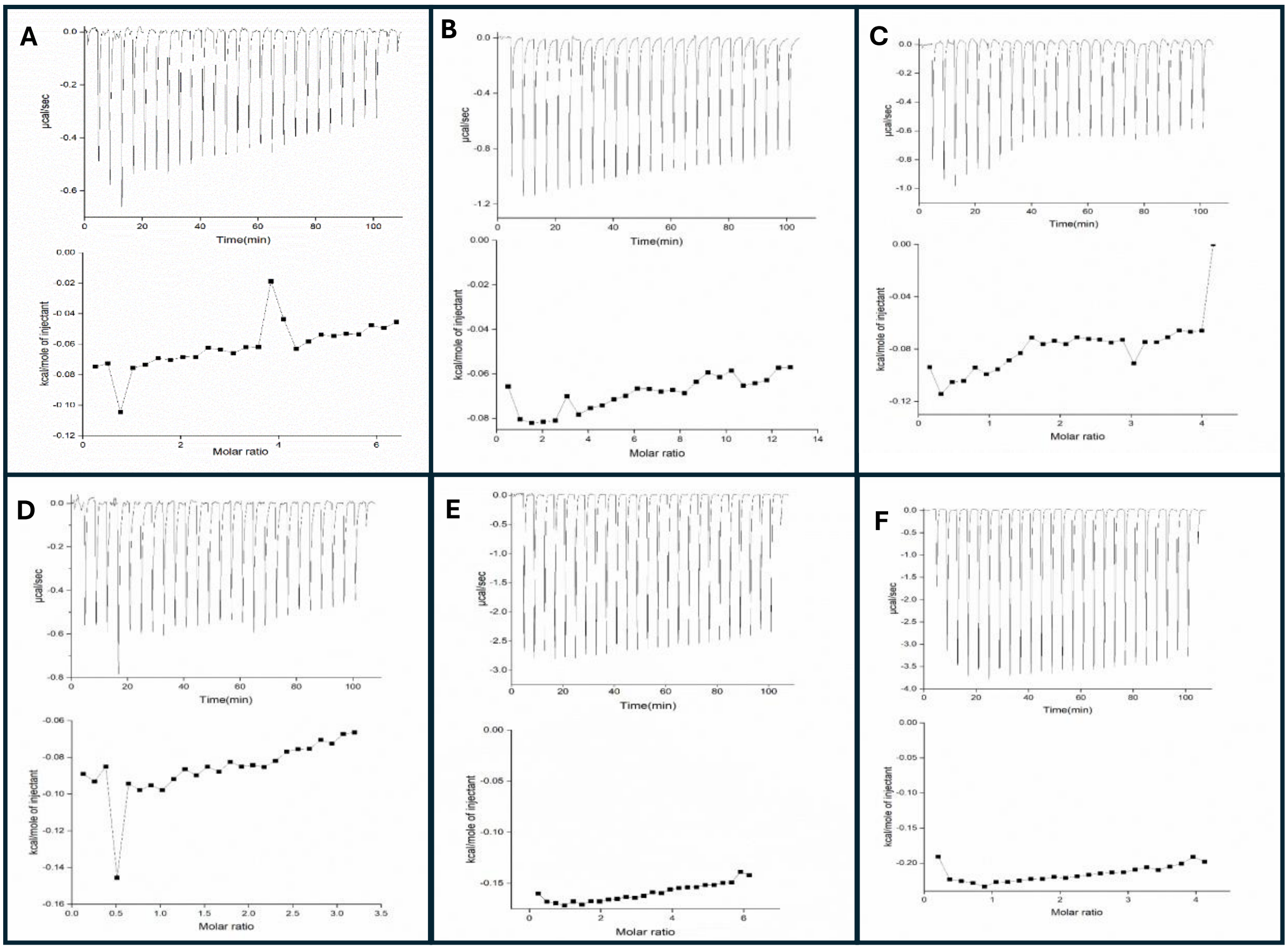


**Figure S2: ITC experiments probing *Er*CRY4a–MTHF interaction at 12 °C across protein and ligand concentrations, Related to Figure 3.** Integrated heats from ITC titrations performed at 12 °C using *Er*CRY4a concentrations of 10–30 µM in the cell and MTHF concentrations of 400 or 800 µM in the syringe. Panels are ordered by increasing *Er*CRY4a concentration and, within each protein concentration, by increasing MTHF concentration: (A) 10 µM *Er*CRY4a / 400 µM MTHF, (B) 10 µM / 800 µM, (C) 16 µM / 400 µM, (D) 20 µM / 400 µM, (E) 20 µM / 800 µM, (F) 30 µM / 800 µM. None of the tested conditions exhibited saturation or sigmoidal curvature indicative of binding. The measured heats were dominated by ligand dilution and closely resembled ligand-to-buffer control experiments. Therefore, data are shown without subtraction to avoid artefacts arising from dilution heats exceeding the total signal.

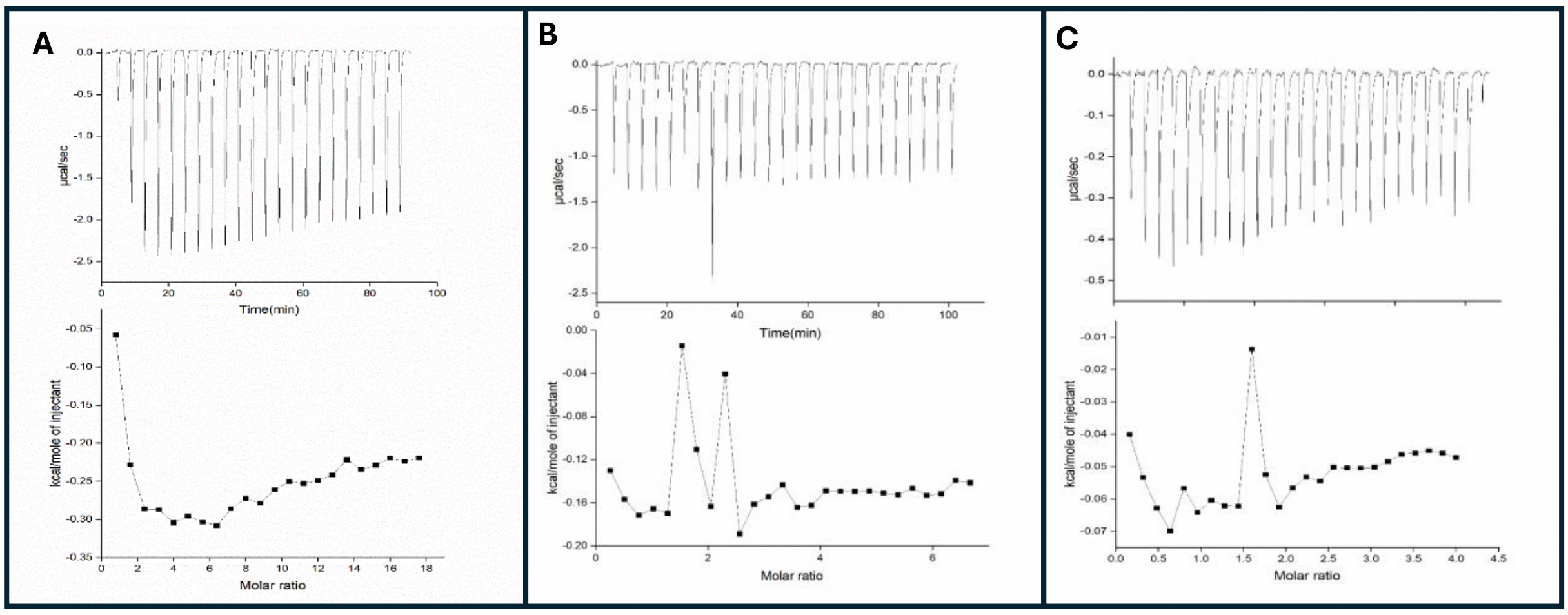


**Figure S3: ITC experiments probing *Er*CRY4a–MTHF interaction at 25 °C across protein concentrations, Related to Figure 3.** Integrated heats from ITC titrations performed at 25 °C using *Er*CRY4a concentrations of 3–16 µM in the cell with a constant MTHF concentration of 400 µM in the syringe. Panels are ordered by increasing protein concentration: (A) 3 µM *Er*CRY4a / 400 µM MTHF, (B) 10 µM / 400 µM, (C) 16 µM / 400 µM. None of the tested conditions displayed saturation behaviour or sigmoidal curvature indicative of binding. As observed at 12 °C, the measured heats were dominated by ligand dilution and were comparable to ligand-to-buffer controls; data are therefore presented without subtraction.

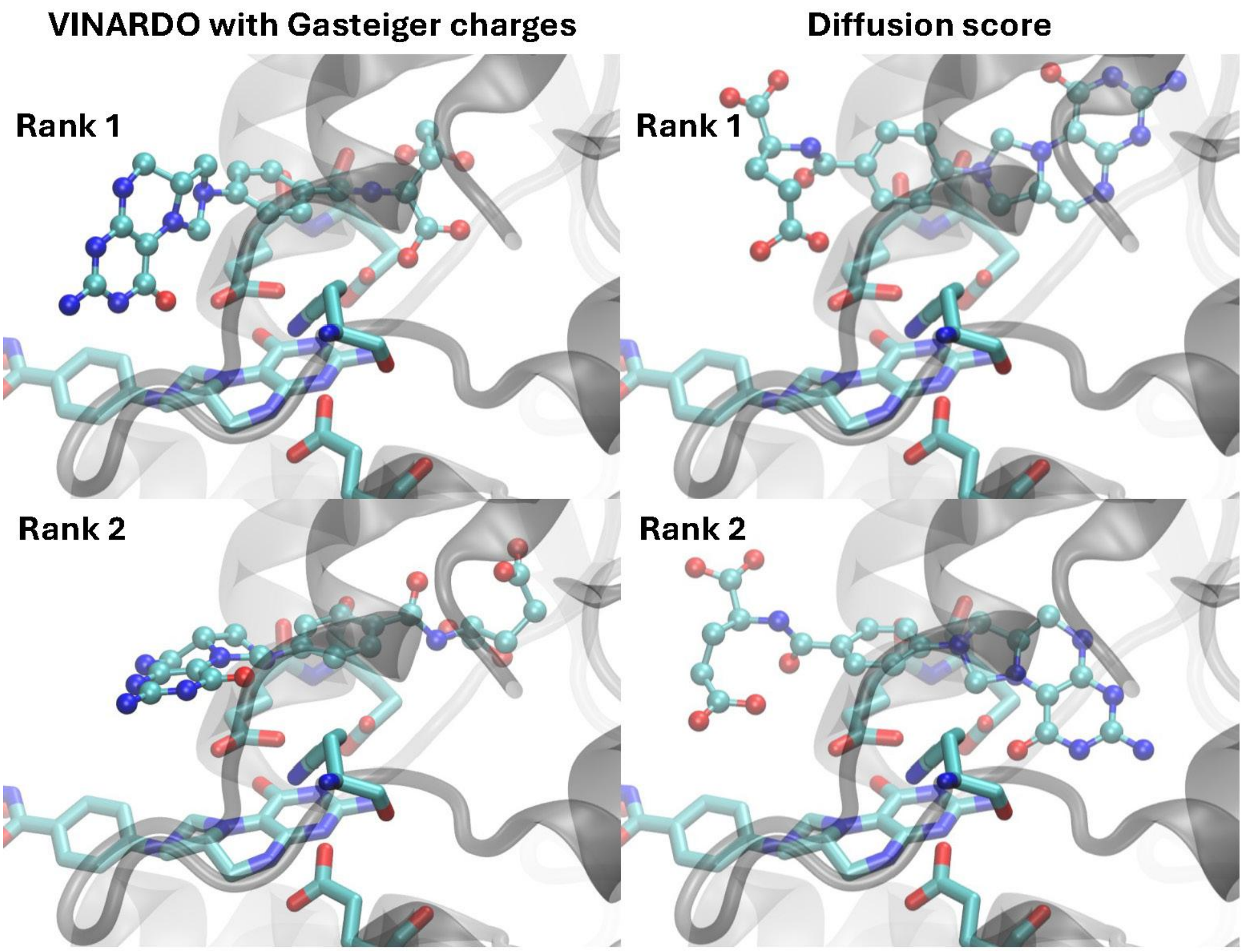


**Figure S4: Testing the Diffusion score as a scoring function for molecular docking, Related to Figure 5.** Top 2 docked ligand conformations (ball-and-stick models) when utilising the VINARDO scoring function with Gasteiger charges (left) or the diffusion score with internal strain and atomistic repulsion from the VINARDO function (right). The secondary structure of the protein is rendered together with licorice representations of the experimentally resolved ligand and some key interaction residues.

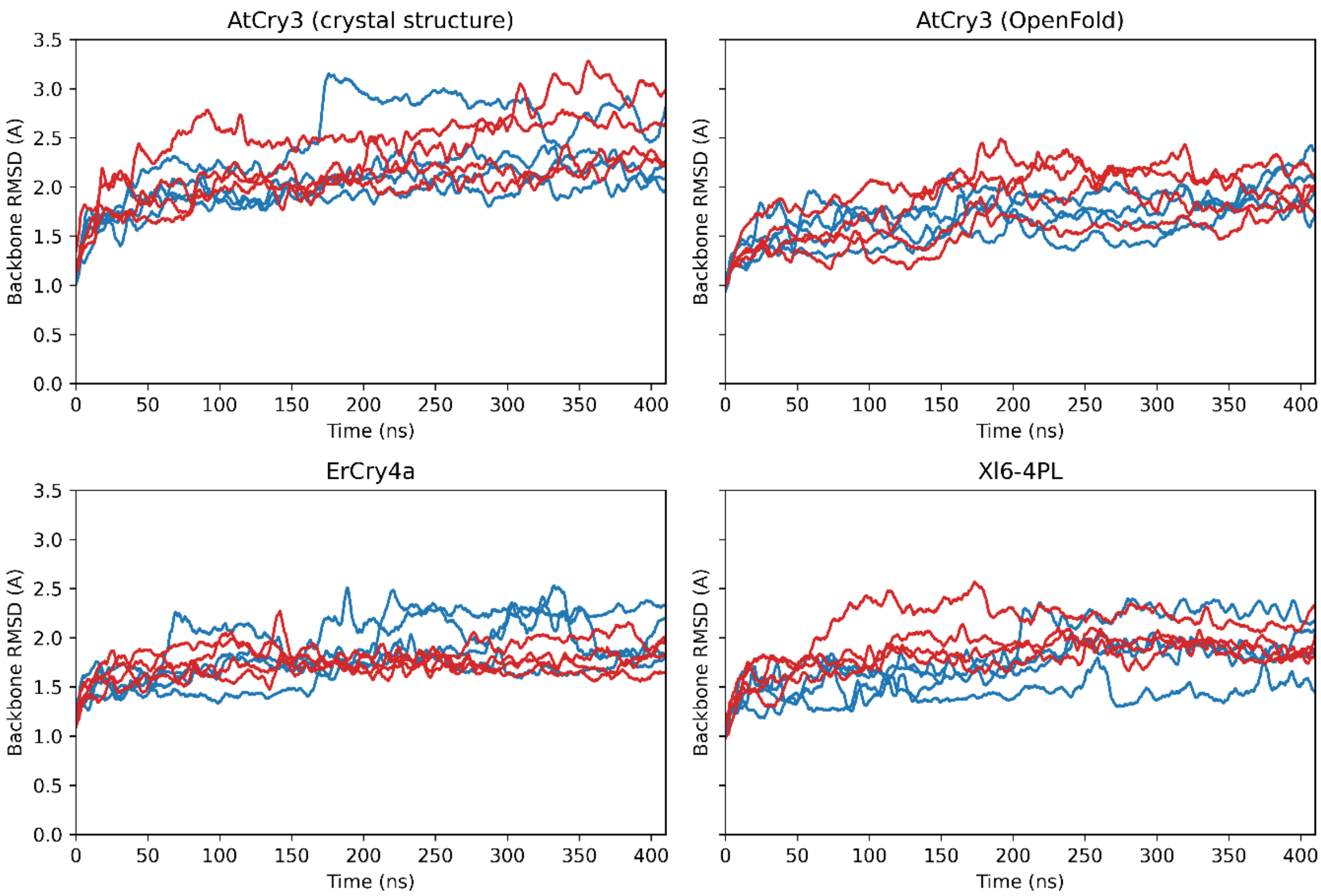


**Figure S5: Backbone RMSD of the considered protein structures, Related to Figure 4 and Figure 5**. The data from four replica simulations at pH 7 (red) and four replica simulations at pH 8 (blue) were calculated with respect to the initial structure and were averaged with a running window of 5 ns width.

**Table S1. Summary of ITC experiments performed to probe *Er*CRY4a–MTHF interaction across temperatures and concentrations, Related to Figure 3.** ITC measurements were conducted at 12 °C and 25 °C over a range of *Er*CRY4a (3–30 µM) and MTHF (400–800 µM) concentrations to maximize the likelihood of detecting a binding isotherm.

| **Temperature** | **[*Er*CRY4a] (µM)** | **[MTHF] (µM)** | **Outcome** |
|---|---|---|---|
| 12 °C | 10 | 400 | no saturation |
| 12 °C | 10 | 800 | no saturation |
| 12 °C | 16 | 400 | no saturation |
| 12 °C | 20 | 400 | no saturation |
| 12 °C | 20 | 800 | no saturation |
| 12 °C | 30 | 800 | no saturation |
| 25 °C | 3 | 400 | no saturation |
| 25 °C | 10 | 400 | no saturation |
| 25 °C | 16 | 400 | no saturation |

**Table S2: Overview of the analyzed properties of the clusters with the most promising binding properties, related to Figure 4 and Figure 5.** Presence: Percentage of protein conformations in which the potential binding site (cluster) contained the docked ligand at least once after all docking attempts. ΔG: Binding score from VINA. Diffusion: Average speed of water molecules when located in the potential binding site (cluster). Normalized SASA: Ratio of molecular surface that is not covered by the protein. Entropic barrier: Measurement of blocked exit pathways out of the cluster position.

| Protein | Ligand | Cluster | Presence % | ΔG (kcal/mol) | Diffusion ($Å^2$/ns) | Normalized SASA | Entropic Barrier |
|---|---|---|---|---|---|---|---|
| *At*CRY3 | MTHF | 3 | 66 | -5.3 | 76 | 0.27 | 0.18 |
| | | 12 | 26 | -5.3 | 50 | 0.25 | 0.18 |
| *Er*CRY4a | MTHF | 3 | 70 | -5.2 | 102 | 0.36 | 0.14 |
| | | 12 | 26 | -5.1 | 95 | 0.34 | 0.15 |
| *Xl*6-4PL | 8-HDF | 1 | 78 | -5.6 | 50 | 0.20 | 0.19 |
| | | 6 | 35 | -5.3 | 38 | 0.20 | 0.19 |
| *Er*CRY4a | 8-HDF | 9 | 21 | -5.6 | 50 | 0.18 | 0.19 |
| | | 19 | 08 | -5.4 | 83 | 0.21 | 0.19 |
